# "Hook"-calibration of GeneChip-microarrays: Theory and algorithm

**DOI:** 10.1186/1748-7188-3-12

**Published:** 2008-08-29

**Authors:** Hans Binder, Stephan Preibisch

**Affiliations:** 1 Interdisciplinary Centre for Bioinformatics, University of Leipzig, D-04107 Leipzig, Germany; 2 Max-Planck-Institute for Molecular Cell Biology and Genetics, D-01307 Dresden, Germany

## Abstract

**Background:**

The improvement of microarray calibration methods is an essential prerequisite for quantitative expression analysis. This issue requires the formulation of an appropriate model describing the basic relationship between the probe intensity and the specific transcript concentration in a complex environment of competing interactions, the estimation of the magnitude these effects and their correction using the intensity information of a given chip and, finally the development of practicable algorithms which judge the quality of a particular hybridization and estimate the expression degree from the intensity values.

**Results:**

We present the so-called hook-calibration method which co-processes the log-difference (delta) and -sum (sigma) of the perfect match (PM) and mismatch (MM) probe-intensities. The MM probes are utilized as an internal reference which is subjected to the same hybridization law as the PM, however with modified characteristics. After sequence-specific affinity correction the method fits the Langmuir-adsorption model to the smoothed delta-versus-sigma plot. The geometrical dimensions of this so-called hook-curve characterize the particular hybridization in terms of simple geometric parameters which provide information about the mean non-specific background intensity, the saturation value, the mean PM/MM-sensitivity gain and the fraction of absent probes. This graphical summary spans a metrics system for expression estimates in natural units such as the mean binding constants and the occupancy of the probe spots. The method is single-chip based, i.e. it separately uses the intensities for each selected chip.

**Conclusion:**

The hook-method corrects the raw intensities for the non-specific background hybridization in a sequence-specific manner, for the potential saturation of the probe-spots with bound transcripts and for the sequence-specific binding of specific transcripts. The obtained chip characteristics in combination with the sensitivity corrected probe-intensity values provide expression estimates scaled in natural units which are given by the binding constants of the particular hybridization.

## 1. Background

The basic mechanism underlying the functioning of DNA microarrays is that of hybridization. Hybridization is defined as the binding between complementary single-stranded nucleic acids. In the case of microarrays one strand is anchored at the surface and the second one is dissolved in solution, referred to as probe and target, respectively. The experimental technique of detecting hybridized probes relies on the fluorescence intensity measurement to infer the transcript abundance specific for a selected gene. The relationship between transcript abundance and intensity is affected by parasitic effects owing to the "technical" variability of repeated measurements and systematic biases which disturb the one-to-one relationship between the input and the output quantity of the measurement [1].

The task of making estimates of the input quantity (transcript concentration) of a measurement from observations of its output (intensity) is called calibration. Calibration of microarray measurements thus aims at removing consistent and systematic sources of variations to allow mutual comparison of measurements acquired from different probes, arrays and experimental settings. Calibration is also called preprocessing because it usually constitutes the first step in the microarray analysis pipeline. It potentially influences the results of all subsequent steps of "higher-level" analyses as well as the biological interpretation of these results, and is therefore a crucial step in the processing of microarray data. The improvement of microarray calibration methods is an essential prerequisite for obtaining absolute expression estimates which in turn are required for the quantitative analysis of, e.g., transcriptional regulation.

Most of the established preprocessing methods rely on algorithms of mainly empirical nature based on the simple assumption of a linear signal response on the transcript concentration in the sample [2-5]. In the last years numerous studies on the physical background of microarray hybridization are published with the perspective of developing improved analysis algorithms [6-12]. For example it has been shown that the probes saturate at higher transcript concentrations which gives rise to a non-linear relation between intensity and transcript concentration. Moreover, benchmark studies have indicated that the proper correction for non-specific background intensity contributions is presumably the most problematic preprocessing step with no satisfactory solution so far.

The immediate aim of most of these papers and also of our previous work [1,13-18], has been to study the physical (and chemical) processes responsible for converting concentrations of specific target RNA of known sequences to measured fluorescence intensities after hybridization. However, the ultimate, still not-achieved aim of these physical approaches has been to provide scientists with feasible calibration methods which estimate absolute specific target concentrations in the presence of a complex non-specific background from fluorescence intensity data.

Proper calibration of microarray data includes several tasks: Firstly it requires the determination of the model describing the basic relationship between the probe intensity and the specific transcript concentration under consideration of relevant parasitic effects which should be straightened out.

Secondly, the magnitude of these effects should be estimated using the intensity information of a given chip or of a series of chips, and, thirdly, one needs practicable algorithms which judge the quality of a particular hybridization and estimate the expression degree from the intensity values.

Moreover, except MAS5 all popular preprocessing methods [2-5] rely on multichip-algorithms for calibration, i.e. they process a series of chips at once together to separate chip- and probe-level effects from each other. The obtained expression measures are consequently context-sensitive and require a minimum number of chips for appropriate data-processing (usually more than four). As a consequence the results are constricted to a particular series of chips, i.e. they depend on the particular selection of chips and require re-calculation upon adding or removing chips. The development of single chip calibration methods is therefore an important additional task to provide virtually context-insensitive expression measures which can be compared between chips and experimental series without reprocessing. This issue requires appropriate metrics for expression measures to enable direct comparison of data from different experiments in consistent units.

This paper addresses these tasks and presents a new single-chip calibration method for microarrays based on a physical model of hybridization. Our so-called hook-method provides a graphical summary of the hybridization characteristics of each microarray which directly transforms into a sort of natural metrics for intensity calibration with the potential to estimate expression values on an absolute scale. This metrics uses mismatched probes on Affymetrix GeneChip arrays as internal reference for judging the hybridization of the perfect matched probes over the whole potential concentration range.

In the first part of the paper we outline the calibration model and validate its relevance using single probe benchmark data. In the second part we apply the model to single chip data and describe the analysis algorithm step by step. Table 1 summarizes the essential notations and symbols used in the paper. Examples which illustrate the performance of the method are presented in the accompanying publication [19].

**Table 1:** Notations

**Superscripts**	Assigns main symbols to...
P = PM, MM	Probe type
h = N, S	Non-specific or specific hybridization
**Subscripts**	Assigns main symbols to...
p, c, set	Probe-, chip- or probe-set-level
0	Values after sequence correction

**Main symbols**	
${\text{I}}_{\text{p}}^{\text{P}}$, M_c_, O_c_	Probe intensity, maximum intensity upon saturation and optical background
$L_{0,p}^{P}$, $L_{p}^{P,h}$	Linearized (de-saturated) intensities
S_set_, N_c_	Specific and non-specific signals
$\Theta_{\text{p}}^{\text{P}}$, $\Theta_{\text{p}}^{\text{P},\text{S}}$, $\Theta_{\text{p}}^{\text{P},\text{N}}$	Probe occupancy: total and due to specific and non-specific hybridization
x_p_^P,S^, x_p_^P,N^	Fraction of specific and of non-specific transcripts among the total amount of bound transcripts
${\text{X}}_{\text{p}}^{\text{P}}$, ${\text{X}}_{\text{p}}^{\text{P},\text{S}}$, ${\text{X}}_{\text{p}}^{\text{P},\text{N}}$	Binding strength: total and due to specific and non-specific hybridization
[S]_p_, [N]_c_	Concentration of specific and non-specific transcript
${\text{K}}_{\text{p}}^{\text{P},\text{S}}$, ${\text{K}}_{\text{p}}^{\text{P},\text{N}}$	Binding constants of a probe for specific and non-specific transcripts
s, n	PM/MM-sensitivity gain for specific and non-specific binding
R	S/N-ratio
Δ, Σ	Hook coordinates
Δ_start_, Σ_start_	Starting values of the hook coordinates
Δ(0), Σ(0)	
Δ(∞), Σ(∞)	End values of the hook coordinates
*α*, *β*	Vertical and horizontal dimensions of the hook curve
*DS*_*p*_	Discrimination score
$\varepsilon_{13}^{\text{P},\text{h}}$(B_p_)	Contribution of middle base B to the signal
$\delta \varepsilon_{\text{k}}^{\text{P},\text{h}}$(b_m_)	Positional dependent sensitivity propfile; k is the start position of sequence motiv b_m _which consists of m adjacent nucleotides
$\delta {\text{A}}_{\text{p}}^{\text{P}}$	Sequence specific contribution to the intensity
${\text{Y}}_{\text{p}}^{\text{P},\text{h}}$	Sensitivity
$p_{p}^{N}$, $p_{c}^{S}$	Density distributions of the non-specific and specific signals
*μ*^*P*^, *σ*^*P*^, *ρ*	Mean value, standard deviation and coefficient of PM/MM-correlation of the normal distributions of the non-specific background signals
*λ*, *λ*_Σ_	decay lengths of the exponential distribution (referring to the S/N-ratio and to the Σ-signal)
*φ*_*c*_	Mean specific signal

**Operation**	
g log(x)	Generalized logarithm
<...>	Arithmetic mean

## 2. Calibration model for microarray data

### The competitive two-species Langmuir model of microarray hybridization

We emphasize on Affymetrix GeneChip microarray data obtained after the chips have been hybridized, scanned and the images have been summarized into hundred-thousands of paired intensity values of perfect match (PM) and of mismatched (MM) probes. The intensities of probe "p" on chip "c" are well described using the Langmuir adsorption isotherm [12,14,20-22],

(1) $$\begin{array}{ccc} {\text{I}}_{\text{p}}^{\text{P}}*={\text{M}}_{\text{c}}\cdot \Theta_{\text{p}}^{\text{P}}+{\text{O}}_{\text{c}} & \text{with} & \Theta_{\text{p}}^{\text{P}}=\frac{{\text{X}}_{\text{p}}^{\text{P}}}{1+{\text{X}}_{\text{p}}^{\text{P}}} \end{array}.$$

Here the superscript denotes the probe-type (P = PM, MM). The indices "p" and "c" assign probe- and chip-specific parameters, respectively. The probe-index implies the chip specificity as well, i.e. p = p, c. This model predicts that the fraction of "occupied", i.e. dimerized oligonucleotides of a probe spot, Θ_p_^P ^(also called surface probe coverage or occupancy), is directly related to the observed intensity, I_p_^P^* [14,18]. The proportionality constant, M_c_, specifies the maximum intensity referring to complete occupancy, Θ_p_^P ^= 1, if all oligonucleotides of the respective probe spot on the given chip are dimerized. The minimum intensity referring to the absence of bound transcripts, Θ_p_^P ^= 0, gives rise to the "optical" background intensity, O_c_. Throughout the paper we will consider only "net" intensities which have been corrected for the optical background before further analysis, ${\text{I}}_{\text{p}}^{\text{P}}={\text{I}}_{\text{p}}^{\text{P}}*-{\text{O}}_{\text{c}}$, using, for example, the zone-algorithm provided by Affymetrix [23].

The surface coverage changes as a hyperbolic function of the "binding strength", X_p_^P^, which additively decomposes into contributions due to specific and non-specific hybridization

(2) $${\text{X}}_{\text{p}}^{\text{P}}={\text{X}}_{\text{p}}^{\text{P},\text{S}}+{\text{X}}_{\text{p}}^{\text{P},\text{N}}.$$

Since the binding strengths follow the mass action law they are related to the concentration of specific and non-specific transcripts, [S]_p _and [N]_c_, respectively, and to the respective effective association constants of duplex formation, K_p_^P,h ^(h = S, N) (see [1] for details),

(3) $${\text{X}}_{\text{p}}^{\text{P},\text{S}}={\left[\text{S}\right]}_{\text{p}}\cdot {\text{K}}_{\text{p}}^{\text{P},\text{S}};{\text{K}}_{\text{p}}^{\text{P},\text{N}}={\left[\text{N}\right]}_{\text{c}}\cdot {\text{K}}_{\text{p}}^{\text{P},\text{N}}.$$

The latter equation assumes that the large number of different non-specific RNA-fragments in the hybridization solution effectively acts like a single species with the common concentration [N]_c _for all probes of the chip [15,16]. Contrarily, the concentration of specific transcripts, [S]_p_, refers to a particular probe sequence, i.e., it represents a "single probe"-property. Microarrays of the GeneChip-type use so-called probe sets of several probes (usually N_set _= 11) for estimating the expression of each considered gene. One expects therefore that all probes of a set probe the same, common transcript concentration, i.e. [S]_set _= [S]_p _for p ∈ set assuming that effects as alternative splicing have been appropriately considered during probe design.

The competitive two-species Langmuir adsorption isotherm (Eq. (1)) considers the effects of non-specific "background" hybridization and of saturation at small and large concentrations of specific transcripts, respectively. The maximum intensity at saturation, M_c_, depends on factors such as the number of oligonucleotides per probe spot (which in turn is related to the density of oligomers and to the spot size), the mean number of optical labels per bound target and the settings of the scanner. These factors affect the PM and MM nearly in the same fashion giving rise to virtually identical values of M_c _at complete saturation of the probe spots under equilibrium conditions (X_p_^P ^>> 1) [16,18].

Recent studies report significantly higher limiting intensity values of the PM, compared with that of the MM, i.e. M^PM ^> M^MM ^[22]. They interpreted this result assuming a probe-dependent partial dissociation of the duplexes during the post-hybridization washing phase. Another, additional explanation might be the truncation of a considerable amount of the probe oligomers due to incomplete synthesis because this effect causes the asymptote-like flattening of the hybridization isotherms at intermediate and large transcript concentrations in a sequence-dependent manner [1,9].

We will apply in the following analysis the special-case of the common intensity asymptote for all probes of the chip according to Eq. (1). Possible consequences of deviations from this assumption for the data analysis will be addressed in a separate study.

### Matched and mismatched microarray probes

The probes on expression microarrays of the GeneChip-type are usually designed in a pairwise fashion. Each probe pair consists of 25-meric PM- and MM-probes where the PM-sequence is assumed to perfectly match a 25-meric section of the target gene. The MM-sequence differs from that of the PM by a single complementary mismatch in the centre of the sequence. The different middle bases of both probes of one pair cause different base pairings in the respective probe/target-duplexes and thus different binding constants (see below and [16]). Let us define the pairwise PM/MM ratio of the binding constants of specific and non-specific hybridization,

(4) $$\begin{array}{ccc} {\text{s}}_{\text{p}}\equiv \frac{{\text{X}}_{\text{p}}^{\text{PM},\text{S}}}{{\text{X}}_{\text{p}}^{\text{MM},\text{S}}}=\frac{{\text{K}}_{\text{p}}^{\text{PM},\text{S}}}{{\text{K}}_{\text{p}}^{\text{MM},\text{S}}} & \text{and} & {\text{n}}_{\text{p}}\equiv \frac{{\text{X}}_{\text{p}}^{\text{PM},\text{N}}}{{\text{X}}_{\text{p}}^{\text{MM},\text{N}}}=\frac{{\text{K}}_{\text{p}}^{\text{PM},\text{N}}}{{\text{K}}_{\text{p}}^{\text{MM},\text{N}}} \end{array},$$

respectively, which specify the noted effect of different base-pairings formed by the PM and MM. For example, the binding strength of the complementary Watson-Crick (WC) base-pairings in the middle of the specific duplexes of the PM exceeds that of the specific duplexes of the MM which form a weaker self-complementary mismatch at this position [15-18]. For the ratio of the specific binding constants one consequently obtains s_p _> 1. Contrarily, for the ratio of the non-specific binding constants one gets n_p _< 1 for purines (Adenine, Guanine) and n_p _> 1 for pyrimides (Thymine, Cytosine) in the middle of the PM sequence owing to the purine-pyrimidine asymmetry of Watson-Crick (WC) base-pair interactions in RNA/DNA duplexes [13,24]. Hence, the parameters s_p _and n_p _specify the PM/MM-affinity gain of a selected probe pair upon specific and non-specific binding, respectively. Both, PM and MM probes obey the hyperbolic adsorption isotherm, Eq. (1) [18]. With Eq. (4) one obtains for the binding strengths of the PM and MM probes

(5) $$\begin{array}{l} \begin{array}{cc} {\text{X}}_{\text{p}}^{\text{PM}}(\text{R})={\text{X}}_{\text{p}}^{\text{PM},\text{N}}\cdot \left(\text{R}+1\right) & \text{and} \end{array}\\ {\text{X}}_{\text{p}}^{\text{MM}}(\text{R})={\text{X}}_{\text{p}}^{\text{PM},\text{N}}\cdot \left(\text{R}/{\text{s}}_{\text{p}}+1/{\text{n}}_{\text{p}}\right) \end{array}$$

Eq. (5) scales the intensity of the PM and MM probes as a function of the relative hybridization degree,

(6) $$\text{R}\equiv {\text{R}}_{\text{p}}^{\text{PM}}=\frac{{\text{X}}_{\text{p}}^{\text{PM},\text{S}}}{{\text{X}}_{\text{p}}^{\text{PM},\text{N}}}=\frac{{\left[\text{S}\right]}_{\text{p}}}{{\left[\text{N}\right]}_{\text{c}}}\cdot \frac{{\text{K}}_{\text{p}}^{\text{PM},\text{S}}}{{\text{K}}_{\text{p}}^{\text{PM},\text{N}}}.$$

This S/N-ratio, R, provides the specific binding strength of the PM in units of the non-specific one. It can serve as a relative measure of the expression degree because it is directly related to the concentration of specific transcripts, [S]_p_. It scales the expression degree in a probe-specific fashion.

**Figure 1:**
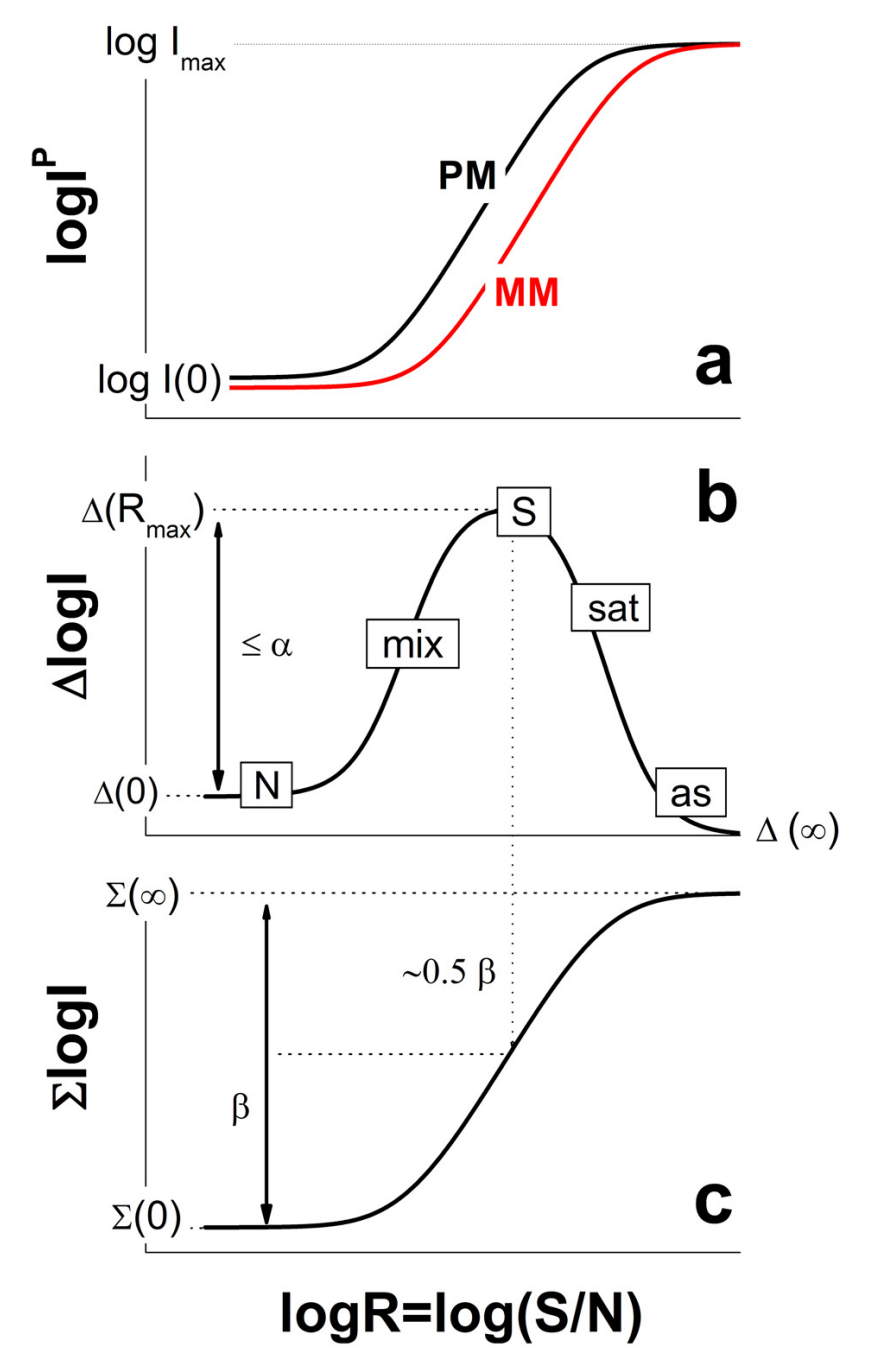
PM- and MM-probe intensities (part a), their log-difference (part b) and -sum (part c) as a function of the specific target concentration (S/N-ratio). The curves are calculated using the Langmuir-model (Eq. (1)). The difference-plot reveals the hybridization regimes of non-specific (N), mixed (mix) and specific (S) hybridization, of saturation (sat) and the asymptotic (as) range. The parameters *α *and *β *used in Eq. (10) specify the data range spanned by the log-difference and -sum.

Part a of Figure 1 shows the courses of the intensities of a typical PM/MM pair as a function of the parameter R (see Eq. (6)). The PM intensity sigmoidally increases from its minimum value, I_p_(R = 0), to I_p_(R = ∞) = M_c_, at small and large abscissa values, respectively. The respective probes referring to these limiting cases are either exclusively non-specifically hybridized or completely saturated with surface coverages of Θ_p_^PM^(0) = X_p_^PM,N^/(1 + X_p_^PM,N^) ≈ X_p_^PM,N ^and Θ_p_^PM^(∞) = 1, respectively. The concentration and S/N-ratio referring to the inflection point of the isotherm at half-way between these values are

(7) $$\begin{array}{ccc} {\left[\text{S}\right]}_{\text{p}}^{50\%}=\frac{1+{\text{X}}_{\text{p}}^{\text{PM},\text{N}}}{{\text{K}}_{\text{p}}^{\text{PM},\text{S}}}\approx \frac{1}{{\text{K}}_{\text{p}}^{\text{PM},\text{S}}} & \text{and} & {\text{R}}^{50\%}=\frac{1+{\text{X}}_{\text{p}}^{\text{PM},\text{N}}}{{\text{X}}_{\text{p}}^{\text{PM},\text{N}}}\approx \frac{1}{{\text{X}}_{\text{p}}^{\text{PM},\text{N}}} \end{array},$$

respectively. They specify the condition at which 50% of the free probes available in the absence of specific transcripts become occupied. The approximations at the right-hand side of Eq. (7) refer to small X_p_^PM,N ^<< 1.

The MM intensity responds in a very similar fashion as that of the PM with increasing R (see part a of Figure 1). The limiting surface coverage of exclusively non-specifically hybridized MM probes at R = 0 is changed compared with that of the PM (see Eq. (4)), Θ_p_^MM^(0) = X_p_^PM,N^/(n_p _+ X_p_^PM,N^) ≈ X_p_^PM,N^/n_p_. The isotherm of the MM is clearly shifted to larger abscissa values in the intermediate R-range owing to the smaller binding strength for specific hybridization (s_p _> 1, see above). For the inflection point of the isotherm one obtains in analogy to Eq. (7)

(8) $$\begin{array}{ccc} {\left[\text{S}\right]}_{\text{p}}^{50\%}={\text{s}}_{\text{p}}\cdot \frac{1+{\text{X}}_{\text{p}}^{\text{PM},\text{N}}}{{\text{K}}_{\text{p}}^{\text{PM},\text{S}}}\approx \frac{{\text{s}}_{\text{p}}}{{\text{K}}_{\text{p}}^{\text{PM},\text{S}}} & ; & {\text{R}}^{50\%}=\frac{{\text{s}}_{\text{p}}}{{\text{n}}_{\text{p}}}\cdot \frac{1+{\text{X}}_{\text{p}}^{\text{PM},\text{N}}}{{\text{X}}_{\text{p}}^{\text{PM},\text{N}}}\approx \frac{{\text{s}}_{\text{p}}}{{\text{n}}_{\text{p}}}\cdot \frac{1}{{\text{X}}_{\text{p}}^{\text{PM},\text{N}}} \end{array},$$

which shows that the horizontal shift between the PM- and MM-isotherms is log(s_p_) and log(s_p_/n_p_) in the log-scale of [S] and R, respectively.

### The delta- and sigma-transformations

The MM probes were designed as reference for estimating the non-specific background contribution to the respective PM intensity [2,5,25]. The "simple" subtraction of the MM-intensity from that of the PM however partly failed as correction method because both probes differently respond to non-specific and specific hybridization due to their complementary middle bases which, for example, gives rise to negative PM-MM intensity differences [15].

According to the Langmuir model, the behaviour of the PM and MM can be understood on the basis of the same hybridization rules where both probe types however differ with respect to their effective association constants for probe/target dimerization (see above). The intensities of the PM and MM are consequently expected to correlate in a well defined fashion. This mutual relation is determined by the mismatch design of the reference probe, the particular probe sequences and by the concentrations of specific and of non-specific RNA fragments in the sample solution used for hybridization on the particular chip [18].

Let us empirically analyze the relation between the PM and MM signals in terms of two simple linear combinations of the log-intensities of a probe pair, namely their difference and average value,

(9) $$\begin{array}{l} \Delta_{\text{p}}\equiv \Delta \log{\text{I}}_{\text{p}}=\log{\text{I}}_{\text{p}}^{\text{PM}}-\log{\text{I}}_{\text{p}}^{\text{MM}}\\ \Sigma_{\text{p}}\equiv \Sigma \log{\text{I}}_{\text{p}}=\frac{1}{2}\left(\log{\text{I}}_{\text{p}}^{\text{PM}}+\log{\text{I}}_{\text{p}}^{\text{MM}}\right) \end{array},$$

(log ≡ log_10 _is the decadic logarithm). The intensity model predicts for this transformation (see Eqs. (1) and (5))

(10) $$\begin{array}{l} \Delta_{\text{p}}(\text{R})=\Delta_{\text{p}}^{\text{start}}+\Delta_{\text{p}}^{\text{Linear}}(\text{R})-\log\left\{\frac{{\text{B}}_{\text{p}}^{\text{PM}}(\text{R})}{{\text{B}}_{\text{p}}^{\text{MM}}(\text{R})}\right\}\\ \text{and}\\ \Sigma_{\text{p}}(\text{R})=\Sigma_{\text{p}}^{\text{start}}+\Sigma_{\text{p}}^{\text{Linear}}(\text{R})-\frac{1}{2}\log\left\{{\text{B}}_{\text{p}}^{\text{PM}}(\text{R})\cdot {\text{B}}_{\text{p}}^{\text{MM}}(\text{R})\right\} \end{array},$$

with the "start", "linear" and the "saturation terms"

$$\begin{array}{ccc} \Delta_{\text{p}}^{\text{start}}=\log{\text{n}}_{\text{p}} & \text{and} & \Sigma_{\text{p}}^{\text{start}}=\log{\text{M}}_{\text{c}}-\beta_{\text{p}} \end{array}$$

$$\begin{array}{ccc} \Delta_{\text{p}}^{\text{Linear}}(\text{R})=\log\left\{\frac{\left(\text{R}+1\right)}{(\text{R}\cdot 10^{-\alpha_{\text{p}}}+1}\right\} & \text{and} & \Sigma_{\text{p}}^{\text{Linear}}(\text{R})=\frac{1}{2}\log\left\{\left(\text{R}+1\right)\cdot \left(\text{R}\cdot 10^{-\alpha_{\text{p}}}+1\right)\right\} \end{array}$$

$$\begin{array}{ccc} {\text{B}}_{\text{p}}^{\text{PM}}(\text{R})=1+10^{-\left(\beta_{\text{p}}-\frac{1}{2}\Delta_{\text{p}}^{\text{start}}\right)}\left(\text{R}+1\right) & \text{and} & {\text{B}}_{\text{p}}^{\text{MM}}(\text{R})=1+10^{-\left(\beta_{\text{p}}+\frac{1}{2}\Delta_{\text{p}}^{\text{start}}\right)}\left(R+10^{-\alpha_{\text{p}}}+1\right) \end{array}$$

respectively. The limiting values of Σ(R) and Δ(R) in the absence of specific transcripts (R = 0) are

(11) $$\begin{array}{l} \begin{array}{ccc} \Delta_{\text{p}}(0)=\Delta_{\text{p}}^{\text{start}}+{\text{o}}_{\Delta } & \text{and} & \Sigma_{\text{p}}(0)=\Sigma_{\text{p}}^{\text{start}}+{\text{o}}_{\Sigma } \end{array}\\ \begin{array}{cccc} \text{with} & {\text{o}}_{\Delta }=\log\frac{1+{\text{X}}_{\text{p}}^{\text{PM},\text{N}}}{1+{\text{X}}_{\text{p}}^{\text{PM},\text{N}}/{\text{n}}_{\text{p}}} & \text{and} & {\text{o}}_{\Sigma }=\frac{1}{2}\log\left(\left(1+{\text{X}}_{\text{p}}^{\text{PM},\text{N}}\right)\cdot \left(1+{\text{X}}_{\text{p}}^{\text{PM},\text{N}}/{\text{n}}_{\text{p}}\right)\right) \end{array} \end{array}.$$

In the limit of weak non-specific binding (X_p_^P,N ^<< 1) the o-terms vanish and the limiting Δ- and Σ-coordinates are given by their start values. The probe-specific exponents in Eq. (10) are defined as

(12) $$\begin{array}{ccc} \alpha_{\text{p}}=\log\frac{{\text{s}}_{\text{p}}}{{\text{n}}_{\text{p}}} & \text{and} & \beta_{\text{p}}=\frac{1}{2}\log{\text{n}}_{\text{p}}-\log{\text{X}}_{\text{p}}^{\text{PM},\text{N}} \end{array}$$

**Figure 2:**
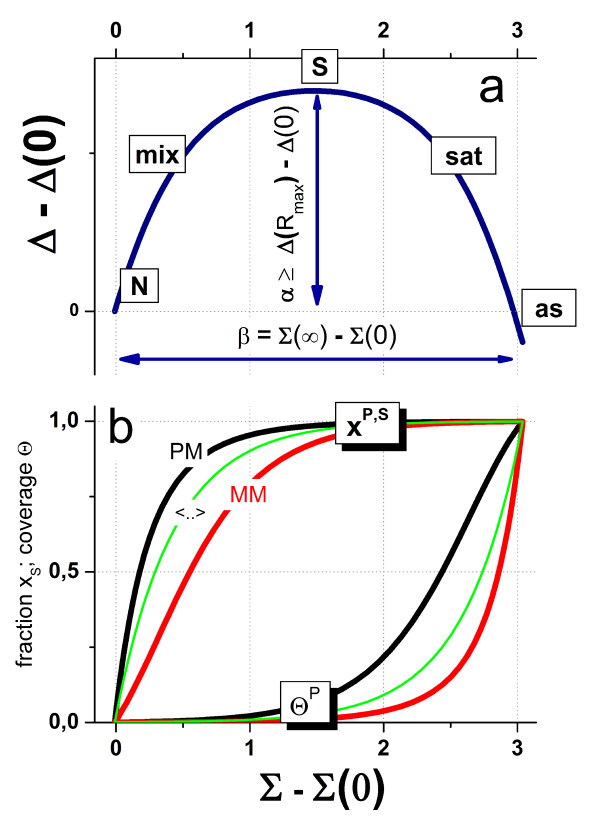
Δ-vs-Σ plot of the PM- and MM-isotherms shown in Figure 1 (part a, see Eq. (9)). The parameters *α *and *β *now specify the height and width of the Δ-vs-Σ trajectory of the PM/MM-probe pair. Part b shows the fraction of specific transcripts bound to PM- and MM-probes (x_S_^P^, P = PM, MM; and their log-mean, <...>) and the total fraction of occupied probe-oligomers (Θ^P^, and of their mean). Note that the fraction of specific transcripts steeply increases in the mix-range whereas the occupancy mainly increases in the sat-range (see Eqs. (17) and (16), respectively).

In summary, the hyperbolic intensity functions of the PM and MM can be transformed into Δ and Σ coordinates which are governed by essentially four parameters, the start values Δ_p_^start ^≅ Δ_p_(0) and Σ_p_^start ^≅ Σ_p_(0) and the exponents *α*_p _and *β*_p_. They were chosen to provide a simple geometrical interpretation of the Δ-vs-Σ trajectory in terms of its start-coordinates and its vertical and horizontal dimension with respect to the start values (see below and Figure 1 and Figure 2).

### The hybridization regimes

Part b and c of Figure 1 show the transformed intensities taken from part a of the figure as a function of the parameter R = R_p_^PM^. The course of the log-difference, Δ_p_(R), can be roughly divided into five regimes which reflect different hybridization characteristics of the PM and the MM probes with increasing degree of specific hybridization (see Figure 1, part b):

(1) **N-regime**: In the non-specific-regime, at small values R → 0, both, the PM and MM nearly exclusively hybridize with non-specific transcripts. Saturation can be typically neglected in this range (B_p_^P ^≈ 1, see Eqs. (10) and (11)). The limiting ordinate value for X_p_^P,N ^<< 1 estimates the ratio of the binding constants referring to the respective pair of complementary middle bases in the PM and MM sequences, Δ_p_(0) ≈ log n_p _(see Eq. (4)). We will use the approximation of weak non-specific binding throughout the paper.

(2) **mix-regime**: In the subsequent mixed-regime, both, specific and non-specific transcripts significantly contribute to the observed intensity of the probes. The log-difference Δ increases with increasing amount of specific transcripts. The positive slope of Δ_p_(R) implies ∂Δ/∂R ~ (1-10^-*α*^) > 0, and thus *α*_p _> 0 or equivalently s_p _> n_p _(see Eq. (12)). The increase of Δ_p_(R) consequently reflects the simple fact that the specific binding constant of the PM exceeds that of the respective MM, i.e., K_p_^PM,S ^> K_p_^MM,S^, if one assumes K_p_^PM,N ^≈ K_p_^MM,N ^(see below and Eq. (4)).

(3) **S-regime**: In the specific-regime the probes predominantly hybridize with specific transcripts. As a consequence, Δ_p _reaches a maximum at $\text{R}={\text{R}}_{\max}\approx 10^{0.5\left(\alpha_{\text{p}}+\beta_{\text{p}}\right)}$ with the ordinate value

(13) $$\Delta_{\text{p}}({\text{R}}_{\max})\approx \Delta_{\text{p}}(0)+\alpha_{\text{p}}-\log\left\{\frac{1+10^{-0.5\left(\beta_{\text{p}}-\alpha_{\text{p}}\right)}}{1+10^{-0.5\left(\beta_{\text{p}}+\alpha_{\text{p}}\right)}}\right\}.$$

This rough approximation assumes Δ_p_(0) << 1 <*β*_p _and R_max _>> 1. At conditions of weak saturation Eq. (13) simplifies with 0.5 (*β*_p _- *α*_p_) >> 1 into $\Delta_{p}\left(R_{\max}\right)\approx \Delta_{p}^{Linear}\left(\infty \right)=\Delta_{p}(0)+\alpha_{p}>0$. At these conditions the height of the maximum directly provides the log-transformed PM/MM-ratio of the specific binding constants, *α*_p_.

(4) **sat-regime**: In the saturation-regime the probes become progressively saturated with bound transcripts (B_p_^P ^> 1). This effect first and foremost affects the PM due to their higher specific binding constant (see above). As a consequence Δ_p _starts to decrease.

(5) **as-regime**: At very large expression degrees both, PM and MM reach their maximum intensity upon complete saturation. In this asymptotic-regime the trajectory reaches the abscissa for R → ∞, Δ_p_(∞) ≈ 0 (see Eq. (10)).

The respective log-sum of the intensities, Σ_p_(R), is shown in part c of Figure 1. It varies in a similar, sigmoidal fashion as the individual log-intensities of the PM and MM (compare part a and c of Figure 1). Here the mix-, S- and sat-regimes merge into one region of increasing Σ whereas the N- and as-regimes provide the minimum and maximum values, $\Sigma_{\text{p}}(0)\approx \Sigma_{\text{p}}^{\text{start}}$ and Σ_p_(∞) = log M_c_, respectively. With Eqs. (11) and (12) one obtains for the difference

(14) *β*_p _≈ Σ_p_(∞) - Σ_p_(0) > 0

*β*_p _specifies the span between the maximum and minimum Σ-values. The Σ-coordinate of the maximum of Δ_p_(R) at R = R_max _becomes

(15) $$\Sigma_{\text{p}}\left({\text{R}}_{\max}\right)\approx \Sigma_{\text{p}}(0)+\frac{1}{2}\beta_{\text{p}}.$$

Eqs. (15) and (14) provide $\Sigma_{\text{p}}\left({\text{R}}_{\max}\right)-\Sigma_{\text{p}}(0)\approx \frac{1}{2}\left(\Sigma_{\text{p}}\left(\infty \right)-\Sigma_{\text{p}}(0)\right)>0$, i.e., the maximum of Δ_*p*_(*R*) roughly bisects the total range of the Σ-coordinate.

### The Δ-vs-Σ trajectory

In the next step we plot the transformed intensities into Δ-vs-Σ coordinates (see Figure 2, part a). This presentation, also known as M-vs-A plot (difference-vs-sum), reflects the binding isotherms of a PM/MM-probe pair. The obtained Δ-vs-Σ trajectory shows a characteristic curved shape with start-, end- and maximum-points referring to the S/N-ratios R = 0, R = ∞ and R = R_max_, respectively. They consequently define the N-, as- and S-hybridization regimes. The mix- and sat-regimes can be attributed to the increasing and decreasing parts of the Δ-vs-Σ trajectory, respectively.

The parameters *α*_p _and *β*_p _define the height and the width of the obtained Δ-vs-Σ curve (see also Eqs. (13) and (14)). The Δ- and Σ-coordinates of the characteristic points depend on the PM/MM-ratios of the binding constants (see Eq. (4)), on the maximum intensity, Σ_p_(∞) = logM_c_, and on the mean intensity of the chemical background due to non-specific hybridization, Σ_p_(0) ∝ log(I_p_^PM^(0)) + log(I_p_^MM^(0)). Hence, the Δ-vs-Σ trajectory links the observed probe intensities with essential hybridization characteristics in terms of simple geometric parameters.

The horizontal scale of the Δ-vs-Σ trajectory

In the Appendix A we show that the difference between the actual Σ-coordinate and its "asymptotic-value", Σ_p_-Σ_p_(∞), estimates the mean probe coverage of the PM and MM probes

(16) $$\langle \Theta_{\text{p}}\rangle =10^{\left(\Sigma_{\text{p}}-\Sigma \left(\infty \right)\right)},$$

whereas the difference between the Σ-coordinate and its "start value", Σ_p_-Σ_p_(0) characterizes the relation between the amount of specific and non-specific hybridization in terms of the fraction of specifically occupied binding sites of the respective probe spot

(17) $$\langle {\text{x}}_{\text{p}}^{\text{S}}\rangle \approx \frac{1-10^{-\left(\Sigma_{\text{p}}-\Sigma_{\text{p}}\left(0\right)\right)}}{1-10^{-\beta_{\text{p}}}}.$$

Eqs. (16) and (17) provide mean values averaged over the respective PM/MM-probe pair. The "individual" coverages of the PM and MM probes, Θ_p_^PM ^and Θ_p_^MM^, and the respective fraction of specifically hybridized oligomers, x_p_^PM,S ^and x_p_^MM,S^, in addition depend on the relative Δ-coordinates Δ-Δ(∞) and Δ-Δ(0), respectively (see Eqs. (42) and (45) in the Appendix A).

Part b of Figure 2 shows the surface coverage and the fraction of specifically occupied oligomers for the Δ-vs-Σ trajectory plotted in part a of the figure. Note that x^P, S ^and Θ^P ^exponentially scale with the coordinate differences Σ-Σ(0) and Σ-Σ(∞), respectively (see Eqs. (17) and (16), respectively).

Consequently, the fraction of specifically occupied probes steeply increases in the raising part of the Δ-vs-Σ trajectory (mix-regime) whereas the probe coverage steeply increases in its decaying part (sat-regime). The contribution of non-specific hybridization and/or the effect of saturation of a particular probe can be essentially neglected if the distance of its Σ-coordinate from the start and/or end points exceeds unity. Particularly, one obtains <x_p_^S^> > 0.9 for Σ-Σ(0) > 1 and < Θ_p_> < 0.1 for Σ(∞)-Σ < 1.

The horizontal shift between the PM-and MM-curves in part b of Figure 2 illustrates the "delayed response" of the MM with respect to the specific transcript concentration: The MM reach a certain ordinate-level of the surface coverage and of the fraction of specifically bound probes at larger abscissa values and thus at larger concentrations of specific transcript concentrations than the PM (see also Eqs. (7) and (8)).

The fraction of specifically bound probes directly transforms into the mean S/N-ratio of the PM and MM (see Appendix A and also Eq. (6)),

(18) $$\langle \text{R}\rangle \approx \frac{10^{\left\{\left(\Sigma_{\text{p}}-\Sigma_{\text{p}}\left(0\right)\right)\right\}}-1}{1-10^{\left\{\left(\Sigma_{\text{p}}-\Sigma_{\text{p}}\left(\infty \right)\right)\right\}}}.$$

For abscissa values Σ < Σ(∞) -1, Eq. (18) simplifies into log(<R> + 1) ≈ Σ-Σ(0). Hence, the Σ-axis nearly linearly scales with the logarithm of the mean S/N-ratio. For the S/N-ratio of the PM, this equation modifies into $\log({\text{R}}_{\text{p}}^{\text{PM}}+1)\approx \left(\Sigma -\Sigma (0)\right)+\frac{1}{2}\left(\Delta -\Delta (0)\right)$ (see Eq. (46) below), i.e., it depends in addition on the vertical coordinate of the Δ-vs-Σ trajectory.

For intermediate abscissa values, Σ(0) + 1 < Σ < Σ(∞) -1, the occupancy of the probe spots (Eqs. (16) and (42)) provide an approximation of the binding strength of specific hybridization of the PM and MM probes (Θ_p_^P ^≈ X_p_^P,S^, see also Eq. (1)) and of their mean

(19) $$\begin{array}{l} \begin{array}{ccc} \log{\text{X}}_{\text{p}}^{\text{PM},\text{S}}\approx -\left(\Sigma_{\text{p}}\left(\infty \right)-\Sigma_{\text{p}}\right)+\frac{1}{2}\Delta_{\text{p}} & ; & \log{\text{X}}_{\text{p}}^{\text{MM},\text{S}}\approx -\left(\Sigma_{\text{p}}\left(\infty \right)-\Sigma_{\text{p}}\right)-\frac{1}{2}\Delta_{\text{p}} \end{array}\\ \begin{array}{cc} \text{and} & \log{\text{X}}_{\text{p}}^{\text{S}}\equiv \frac{1}{2}\left(\log{\text{X}}_{\text{p}}^{\text{PM},\text{S}}+\log{\text{X}}_{\text{p}}^{\text{MM},\text{S}}\right)\approx -\left(\Sigma_{\text{p}}\left(\infty \right)-\Sigma_{\text{p}}\right) \end{array} \end{array}.$$

In summary, the position of a probe-point along the Σ-coordinate estimates the hybridization degree of the respective probe spot in terms of relative concentration measures characterizing either the S/N-ratio (Eq. (18)), the relative occupancy of the probe oligomers with specific transcripts (Eq. (17)), their overall degree of occupancy of (Eq. (16)) and the specific binding strength of the considered probe pair (Eq. (19)).

**Figure 3:**
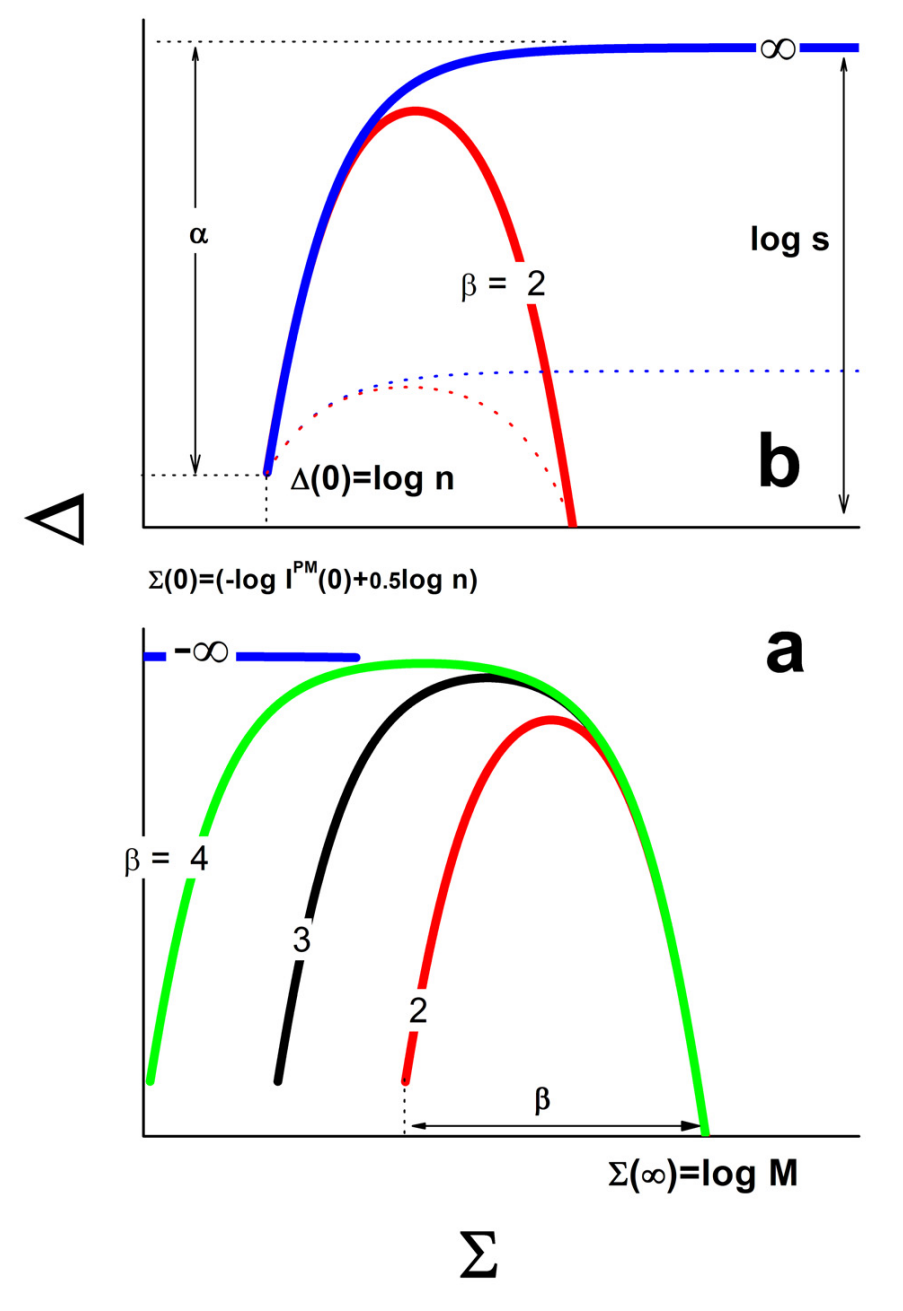
Part a: Δ-vs-Σ trajectories upon decreasing contribution of the non-specific background. The width of the curve, *β*, defines the logarithm of the binding strength of non-specific hybridization in the absence of specific transcripts (Eq. **(12)**). Part b shows two trajectories of different height. The PM/MM-gain parameter s defines the height in the absence of saturation. The narrowing of the trajectories decreases the maximum (see Eq. **(13)**).

The probe coverage (Eq. (16)) provides an additional interpretation of the horizontal dimensions of the Δ-vs-Σ trajectory: For the N-point one obtains with Σ = Σ(0) the coverage due to non-specific hybridization, $\langle \Theta_{\text{p}}^{\text{N}}(\text{R}=0)\rangle \approx 10^{\left(\Sigma_{\text{p}}(0)-\Sigma_{\text{p}}(\infty )\right)}=10^{-\beta_{\text{p}}}$, because (almost) exclusively non-specific transcripts bind to the probes. Note that this "non-specific" coverage is exponentially related to the "width"-exponent, *β*_p _(see Eq. (14)), and thus to the horizontal distance between the N- and the as-points. The remaining, not-occupied and thus free oligomers serve as potential binding sites for specific targets, i.e., $\langle \Theta_{\text{p}}^{\text{free}}(\text{R}=0)\rangle =1-10^{-\beta_{\text{p}}}$. The horizontal dimension of the Δ-vs-Σ trajectory consequently specifies the maximum amount of free probes available for specific binding at R = 0 and thus the measurement range of the probe spots for estimating the expression degree. The narrowing of the model curves reflects the diminishing capacity of the respective probes for specific transcript binding. Figure 3 (part a) illustrates the narrowing of the Δ-vs-Σ trajectory upon increasing the non-specific background contribution. The special ideal case *β *= -∞ consequently refers to hybridization without non-specific background.

### The vertical scale of the Δ-vs-Σ trajectory

The Δ-coordinate of a probe is directly related to the so-called discrimination score DS used by Affymetrix as a relative measure of the PM-MM intensity difference.

(20) $$\begin{array}{ccc} \Delta_{p}=\log\frac{1+DS_{p}}{1-DS_{p}}\approx \frac{2}{\ln10}\cdot DS_{p} & with & DS_{p}=\frac{I_{p}^{PM}-I_{p}^{MM}}{I_{p}^{PM}+I_{p}^{MM}} \end{array}.$$

The discrimination score roughly estimates the fraction of the signal due to specific hybridization (see [26]). The approximation on the right hand side of Eq. (20) seems save for small values DS << 1.

The discrimination score serves as the basic parameter in the MAS5-algorithm to calculate the so-called detection call (DC) which judges the "presence" or "absence" of a gene. Hence, the vertical scale of the Δ-vs-Σ trajectory is related to the detection call: the higher the Δ_p_-value of a probe the higher the probability of the presence of the respective specific transcript in the hybridization solution. We will discuss this point more in detail in the accompanying paper in connection with our alternative method for classifying the genes into present and absent ones (see below).

The vertical scale of the Δ-vs-Σ trajectory admits an additional interpretation in terms of different strengths of the base pairings of the PM and MM probes. Particularly, the Δ-coordinates of the N- and the S-points estimate the ratio of the binding constants of the PM and MM upon specific and non-specific hybridization according to Eqs. (4), (11) and (13). We have previously shown that the log-ratio of the binding constants of the PM and MM probes can be interpreted in terms of the effective free energy difference for duplex formation with the respective targets [15,16,18]. For the MM-design used for GeneChip expression arrays it roughly refers to the effective free energy change upon replacement of the Watson Crick (WC) pairing in the middle position of the probe/target duplexes with the respective self complementary (SC) pairing in the specific duplexes and with the complementary WC-pairing in the non-specific duplexes, respectively, i.e.,

(21) $$\begin{array}{l} \begin{array}{ccc} \log{\text{s}}_{\text{p}}\approx -\Delta \varepsilon_{13}^{\text{WC}-\text{SC}}({\text{B}}_{\text{p}}) & \text{and} & \log{\text{n}}_{\text{p}}\approx -\Delta \varepsilon_{13}^{\text{WC}-\text{WC}}({\text{B}}_{\text{p}}) \end{array}\\ \text{with}\\ \begin{array}{ccc} \Delta \varepsilon_{13}^{\text{WC}-\text{SC}}({\text{B}}_{\text{p}})\equiv \left(\varepsilon_{13}^{\text{PM},\text{S}}({\text{B}}_{\text{p}})-\varepsilon_{13}^{\text{MM},\text{S}}({\text{B}}_{\text{p}}^{\text{c}})\right) & \text{and} & \Delta \varepsilon_{13}^{\text{WC}-\text{WC}}({\text{B}}_{\text{p}})\equiv \left(\varepsilon_{13}^{\text{PM},\text{N}}({\text{B}}_{\text{p}})-\varepsilon_{13}^{\text{MM},\text{N}}({\text{B}}_{\text{p}}^{\text{c}})\right) \end{array} \end{array}.$$

Here Δ*ε*_13_^WC-SC ^denotes the dimensionless free energy gain (given in units of the thermal energy, RT) upon replacements of the type B•b^c ^→ B^c^•b^c ^(i.e. WC → SC) for the base B_p _= A, T, G, C at sequence position 13 of the probe (for example, C•g → G•g; upper case letters refer to the DNA-probe; lower case letters refer to the bound RNA-fragment, b = a, u, g, c; the superscript "c" indicates the respective complement). Accordingly, Δ*ε*_13_^WC-WC ^is the respective free energy change upon WC-reversals, B•b^c ^→ B^c^•b (for example, C•g → G•c).

Hence, the ordinate position of the starting point of the Δ-vs-Σ trajectory estimates the effective free energy change upon replacing the central base in complementary WC-pairings, i.e. Δ_p_(0) ≈ -Δ*ε*^WC-WC^(B_p_) (see Eqs. (11) and (21)). The relative ordinate value of the maximum is related to the respective free energy change upon replacing the central WC-pairing in the specific PM-duplexes by the respective SC-pairing in the MM-duplexes, i.e. Δ_p_(R_max_) ≈ -Δ*ε*^WC-SC^.

Figure 3 illustrates that the maximum height of the Δ-vs-Σ trajectory starts to decrease for relatively small widths referring to large strengths of non-specific hybridization (*β*_p _< 3) because saturation onsets almost in the mix-range. In such cases the observed vertical dimension of the trajectory potentially underestimates the height-parameter *α*_p _(see Eq. (13)) which however can be obtained by appropriate curve fitting using Eq. (10) (see below).

In summary, the Δ-vs-Σ trajectory spans a sort of natural or intrinsic metric system between distinctive points which characterizes the binding thermodynamics of the probes of the particular microarray. The horizontal dimension characterizes the measurement range of the respective probe whereas the vertical dimension reflects the free energy gain due to the change of the central base pairing in the respective duplexes of the PM and MM probes.

### Δ-vs-Σ trajectories of individual probes

Each probe is characterized by its "individual" Δ-vs-Σ trajectory which describes the intensity change upon increasing content of S-transcripts in the range 0 ≤ R ≤ ∞. We used the Affymetrix HG-133 spiked-in data-set to study the R-dependence of selected probes . This data set was generated by Affymetrix to calibrate the observed intensities on the basis of known transcript concentrations. Particularly, transcripts referring to 42 selected genes were titrated with increased concentration onto a series of chips using the Latin-squares design. The non-specific background was taken into account by adding a HeLa-cell line extract to all hybridizations which does not contain the spiked-in transcripts.

**Figure 4:**
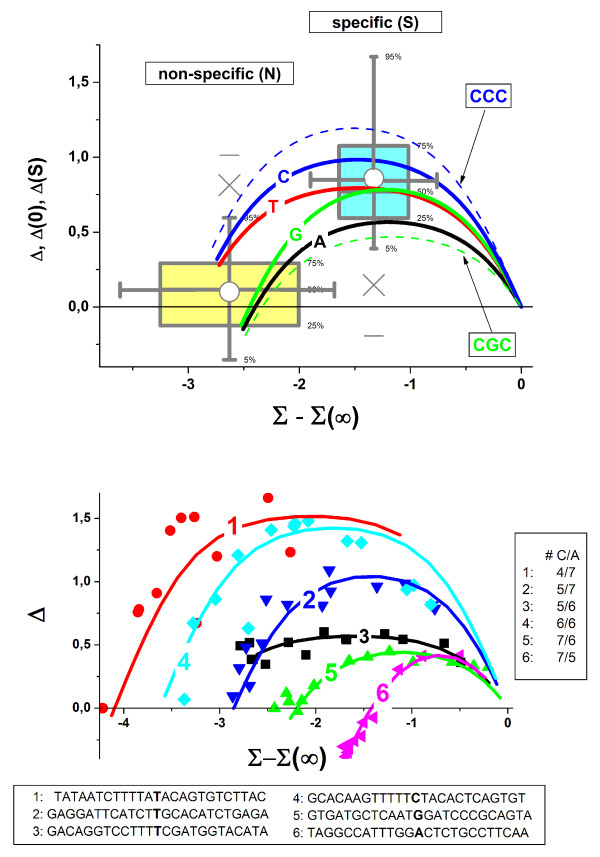
Upper panel: Δ-vs-Σ trajectories of six selected probe-pairs taken from the HG-U133 spiked-in experiment (Affymetrx). The sequences of the PM-probes are shown in the insertion below. The probe-pairs are numbered with increasing C and decreasing A content of the respective PM-probe (see of insertion on the right). The symbols are the experimental data. The lines are calculated using Eq. **(10)**. Lower panel: Variability of the Δ-vs-Σ trajectories of all 498 spiked-in probes. The boxplot indicates the scattering of the start- and maximum-coordinates for R = 0 and R = R_max_, respectively. The Δ-vs-Σ trajectories are averaged over all probe-pairs with the middle base A, T, G and C of the PM, respectively. The variability due to the middle base refers to the 75%/25% quantiles of the distribution of the individual probe trajectories. The consideration of the nearest neighbours in terms of the middle triple extends this range: only the trajectories with the largest and smallest maximum value (for middle triples CCC and CGC, respectively) are shown (see also ref. [18]).

Part a of Figure 4 shows the trajectories of six selected probes together with fits by means of Eq. (10) (compare curves and symbols). The probe-labels 1 to 6 are chosen to increase with increasing number of C and decreasing number of A per probe sequence (see Figure 4). The observed intensities and thus also the trajectories are functions of the binding constants for DNA/RNA duplex formation, which in turn depend on the sequences of the 25 meric probes. For example, the binding affinity of C•g WC-pairings exceeds that of A•u pairs in the hybrid duplexes. In general, the probes with a higher amount of cytosines are therefore expected to bind the RNA fragments more strongly than probes with a higher amount of adenines. Equation (3) predicts for the increase of K_p_^P,N ^(and of logX_p_^PM,N^, see Eq. (3)) the decrease of the horizontal dimensions, *β*_p_, of the respective probe-trajectory.

Indeed, the increase of the cytosine-content causes the narrowing of the trajectories by the shifting of their start-point, Σ_p_(0), towards larger abscissa values at invariant Σ_p_(∞) = const., which is assumed to be constant across all probes because of their common maximum binding capacity. Note that the width of the trajectories and thus the binding strength of the non-specific background varies over about two orders of magnitude, logX_p_^PM,N ^≈ -4 to -2, for the six selected probes.

The Δ-coordinates of the starting- and maximum-points of the selected probe-trajectories show considerable variation without obvious correlation to their sequence characteristics. We calculated the trajectories of all spiked-in probes (~500) using the results of our previous analysis of the hybridization isotherms (see refs. [18] and [16] for details) to estimate the variance of the positions of their starting- and maximum-points. The boxplot in part b of Figure 4 visualizes the center and the width of the distributions of the obtained Δ_p_(0)- and Δ_p_(R_max_)-data in vertical and horizontal directions.

The respective coordinates of the individual probe-trajectories depend mainly on the particular probe pairings of the middle bases in the non-specific and specific duplexes, respectively (see Eqs. (11) – (13) and (21)). To filter out the underlying sequence effects we calculated "mean" trajectories for all probe pairs with a certain middle base (see Figure 4, part b). These middle-base related mean trajectories are shifted each to another in vertical direction according to C ≈ T > G ≈ A for the N-, and C > G ≈ T > A for the S-point, respectively. This systematic trend is in agreement with Eq. (21) which predicts that the vertical positions of the N- and S-points are functions of the middle base of the respective probe sequences. The observed relations reflect the purine-pyrimidine asymmetry of binding strength of complementary WC-pairings at the N-point (i.e., Δ*ε*_13_^WC-WC^(B) ≠ Δ*ε*_13_^WC-WC^(B^c^)) and the higher stability of the WC-pairings compared with SC-mismatches at the S-point, Δ*ε*_13_^WC-SC^(B) (see Eq. (21)). Note that the specific binding constants of PM exceed that of the MM on the average by the factor of s ≈ 7 whereas for non-specific binding one obtains a mean ratio of n ≈ 1.2.

The comparison of the middle-base related trajectories with the width of the N- and S-boxes indicates that the systematic effect due to the middle-base explains the variability of the Δ_p_(0)- and Δ_p_(R_max_)-coordinates in the limits of their 25% and 75% quartiles. The consideration of the nearest neighbors of the middle base further broadens this range: For illustration we show the respective mean trajectories for the "middle triples" CCC and CGC which provide the strongest and weakest binding affinities among the 64 possible combinations of three adjacent bases, respectively (see [18] and [13]).

In summary, the transformed intensity data of individual probes are well described by the Δ-vs-Σ trajectories predicted by the Langmuir-isotherms. The presented data illustrate the probe-specific variability of the Δ-vs-Σ trajectories due to sequence effects. The positions of the start- and maximum-points can be attributed to the differences between the PM and MM probe-sequences which affect the respective binding constants in a middle-base dependent fashion.

## 3. The hook-algorithm for single-chip calibration

The Δ-vs-Σ trajectories describe the behaviour of PM/MM-probe intensities as a function of the RNA-concentration on a relative scale. The analysis of probe-specific trajectories seems not applicable for probe data which are taken from a single chip because each probe pair refers exactly to only one concentration and thus to only one point along its "individual" Δ-vs-Σ trajectory. On the other hand, the large number of probes per chip (and the presence of considerable amounts of specific targets) lets us suggest that their hybridization degrees usually cover the whole potentially possible concentration range. Our idea is to characterize the performance of a particular hybridization experiment in terms of its mean Δ-vs-Σ trajectory averaged over all probes of one particular microarray in analogy to the "individual" Δ-vs-Σ trajectory of each single probe. The horizontal and vertical dimensions of this mean Δ-vs-Σ trajectory are expected to provide the hybridization metrics of the considered chip in terms of characteristic concentration measures (e.g. the mean level of non-specific background or the mean occupancy and saturation level of the probe spots) and of characteristic effective free energy differences due to the used mismatch-design of the probe pairs.

### The raw hook curve

**Figure 5:**
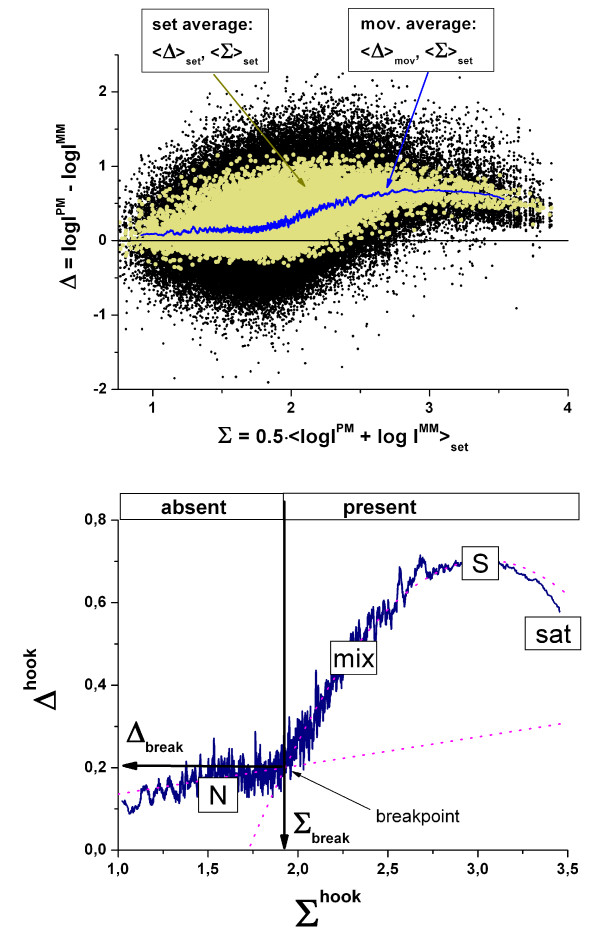
Upper panel: Δ-vs-<Σ>_set _plot of the intensity data of one complete chip taken from the HG-U133 spiked-in experiment (Affymetrix): Probe intensity data (black dots), probe-set log-averaged data (light dots) and moving average over ~100 probe-sets (line). The latter plot is enlarged in the lower panel: It is called (raw) hook curve because of its characteristic shape. The N-, mix-, S- and sat-hybridization regimes are indicated (compare with Figure 2). The breakpoint is used to classify the probesets into absent and present ones (see text).

To construct the mean Δ-vs-Σ trajectory we plot the PM-MM log-intensity difference of all probe pairs of a particular chip, Δ_p_, versus the set-averaged log intensity of the respective probe set, <Σ>_set_, in a first step (see Eq. (9) and part a of Figure 5). Additional set-averaging of the log-difference, <Δ>_set_, reduces the scatter width of the data points along the ordinate roughly by the factor of ~ √N_set _~ 3 (see the yellow dots in Figure 5). In the next step we smoothed these data by calculating the moving average over a sliding window of N_mov _≈ 100 subsequent probe sets along the abscissa to extract the mutual dependence between <Δ>_set _and <Σ>_set_. The resulting plot is called (raw-) hook-curve because of its typical shape (see part b of Figure 5). Each probe-set is characterized by its "hook"-coordinates, Σ^hook ^= <Σ>_p∈set _and Δ^hook ^= <<Δ_p_>_p∈set_>_mov_.

The shape of the hook curve basically agrees with that of the Δ-vs-Σ trajectory (compare with Figure 2). We attribute the rising and decaying part and the maximum in-between to the mix-, sat- and S-regimes, respectively, which refer to the mean hybridization level of the respective probes on the chip. The hook-curve obviously does not reach the asymptotic as-regime with Δ(∞) ≈ 0. This result does not surprise because complete saturation is usually circumvented by reasonably chosen hybridization conditions. Otherwise the probes completely lose their sensitivity to detect changes of the transcript concentration [14].

At small abscissa values the hook curve starts with a virtually horizontal part (slope(N) < 0.1) which is separated from the subsequent mix-range (slope(mix) > 0.6) by a distinct break-point. In the appendix we show that the observed initial tiny slope can be explained by the relative strong correlation between the intensities of non-specifically hybridized PM- and MM-probes in the absence of specific transcripts (*ρ *> 0.7, see Eq. (49)) which in turn seems reasonable in view of the nearly equal sequences of both probe-types which suggest similar variations of the sequence-specific bindings strengths from probe pair to probe pair. On the other hand, for the mix-region the hybridization model predicts a considerably increased slope of slope(mix) ≈ 1.15·*α*_p _≈ 0.9 > slope(N) ≈ 0.8 (see Eqs. (47) and (49) with·*α *> 0.8 and *ρ *> 0.7, respectively). Hence, the break in the course of the hook-curve can be explained by the onset of specific hybridization for probes (and probe-sets) located on the right from this point.

Accordingly, the break-point between the N- and mix-ranges was used to classify the probe-sets into two sub-ensembles: (i) "absent"-ones for Σ^hook ^< Σ^break^, which are assumed to hybridize predominantly non-specifically owing to the absence of specific transcripts in the hybridization solution (R = 0); and (ii) "present"-ones for Σ^hook ^> Σ^break^, which significantly hybridize specifically owing to the presence of specific transcripts (R > 0).

The exact position of the break point of a particular hook curve was estimated by a simple algorithm based on the joint least-squared fit of the Δ^hook^-data to a linear function for Σ^hook ^< Σ^break^, and a quadratic function for Σ^hook ^> Σ^break ^(see Figure 5). The algorithm systematically varies the position of the break, Σ^break ^finally returning the optimum value of Σ^break ^which best fits the data.

### Sensitivity-corrected intensity-data and sensitivity profiles

The intensity of a probe and thus also its (Δ, Σ)-coordinates depend on the concentration of RNA-transcripts and on the binding constants for specific and non-specific hybridization as well (see Eq. (5)). These constants are functions of the respective probe sequences giving rise to the scattering of the individual probe intensities over one-to-three orders of magnitude [14]. This variability is not related to changes of the transcript concentration, and thus it introduces considerable uncertainty if one aims at interpreting the (Σ_set_^hook^, Δ_set_^hook^)-coordinates of a probe in terms of its hybridization degree. In the next step we therefore correct the intensities for probe specific effect according to

(22) $$\begin{array}{l} \begin{array}{cc} \log{\text{I}}_{0,\text{p}}^{\text{P}}=\log{\text{I}}_{\text{p}}^{\text{P}}-\delta {\text{A}}_{\text{p}}^{\text{P}} & \text{with} \end{array}\\ \delta {\text{A}}_{\text{p}}^{\text{P}}=(1-{\text{x}}_{\text{p}}^{\text{P},\text{N}})\cdot \delta {\text{A}}_{\text{p}}^{\text{P},\text{S}}+{\text{x}}_{\text{p}}^{\text{P},\text{N}}\cdot \delta {\text{A}}_{\text{p}}^{\text{P},\text{N}} \end{array},$$

where I^P^_0,p _denotes the corrected intensity of probe p. The sequence-specific incremental contribution, *δ*A_p_^P ^(P = PM, MM), the so-called sensitivity of the probe, additively splits into two terms due to specific and non-specific hybridization, *δ*A_p_^P,N ^and *δ*A_p_^P,S^, which are weighted by the fraction of non-specifically and specifically hybridized oligomers, $x_{p}^{P,N}=\min(L_{p}^{P}(0)/L_{p}^{P},1)$ and x_p_^P,S ^= (1 - x_p_^P,N^) with logL_p_^P^(0) ≈ logI_p_^P^(0) = <logI_p_^P^>_p∈N _+ *δ*A_p_^P,N ^and L_p_^P ^= I_p_^P^/(1-I_p_^P^/M_c_), respectively. The brackets <...>_p∈N _denote averaging over all probes from the N-range of the hook curve.

The intensity-correction according to Eq. (22) requires the knowledge of two sensitivity-values for each PM and each MM probe, *δ*A_p_^P,S ^and *δ*A_p_^P,N ^(P = PM, MM), respectively. They were estimated using either the so-called single-nucleotide (SN, m = 1), the nearest neighbour (NN, m = 2) or the next to nearest neighbour (NNN, m = 3) model. This approach additively decomposes the increments *δ*A_p_^P,h ^into positional and sequence-dependent sensitivity terms, *δε*_k_^P,h^(b_m_) (m = 1,2,3) referring either to single nucleotides (b_1 _= B), to adjacent duplets (b_2 _= BB') or triplets (b_3 _= BB'B"; B,B',B"' = A, T, G, C; see also [27]),

(23) $$\delta {\text{A}}_{\text{p}}^{\text{P},\text{h}}=\sum_{\text{k}=1}^{25-\text{m}+1}\sum_{{\text{b}}_{\text{m}}}\left(\delta \varepsilon_{\text{k}}^{\text{P},\text{h}}({\text{b}}_{\text{m}})\cdot \delta_{\text{k}}({\text{b}}_{\text{m}},\xi_{\text{k},\text{m}}^{\text{P}})\right),$$

with *δ*_k_(b_m_, *ξ*^P^_k,m_) = 1 for b_m _= *ξ*^P^_k,m _and *δ*_k_(b_m_, *ξ*^P^_k,m_) = 0, otherwise. Here *ξ*^P^_k,m _denotes a subsequence of m consecutive bases starting at position k of the respective probe sequence. Each set of sensitivity terms consequently comprises 25 × 4 = 100, 24 × 16 = 384 and 23 × 64 = 1472 parameter values for the SN, NN and NNN models, respectively.

Altogether, the intensity-correction requires four sets of such profiles, *δε*_k_^P,h^(b_m_), referring to P = PM, MM and h = N, S, respectively. We used weighted multiple linear regression of the normalized intensity data taken from selected sub-ensembles of probe sets to estimate the required sensitivity-profiles (see Appendix C and [18]). These sub-ensembles were chosen to comprise probes which are predominantly hybridized with non-specific (p ∈ N) or specific (p ∈ S) transcripts for estimating *δε*_k_^P,N^(b_m_) and *δε*_k_^P,S^(b_m_), respectively. Probes of the former sub-ensemble are taken from the initial horizontal part of the hook curve which was assigned to the N-regime meeting the condition Σ_p_^hook ^< Σ^break ^(see Figure 5). The respective absent-probes are assumed to bind predominantly non-specific transcripts.

The second sub-ensemble of predominantly specifically hybridized probes (p ∈ S) was selected according to the condition Σ_set_^hook ^> Σ^80% ^where Σ^80% ^defines a threshold referring to <x^S^> > 0.8, i.e. to probe spots with a fraction of specifically-hybridized oligomers of at minimum 80%. According to Eq. (17) the respective probes are selected to meet the "horizontal distances"-criterion with respect to the break-point (Σ^80% ^- Σ^break^) > -log(0.2) ≈ 0.7. The somewhat arbitrary choice of <x^S^> > 0.8 turned out to be not very crucial with respect to the quality of the obtained correction. On one hand the higher the value of <x^S^> the smaller the residual contribution of non-specific hybridization in the selected sub-ensemble and the better the obtained sensitivity profiles characterize specific binding [16]. On the other hand, the number of probe sets in the sub-ensemble decreases with increasing <x^S^> giving rise to more noisy sensitivity profiles and thus to less precise correction terms [16]. The chosen value of <x^S^> >0.8 provides a good compromise (see Figure 5).

**Figure 6:**
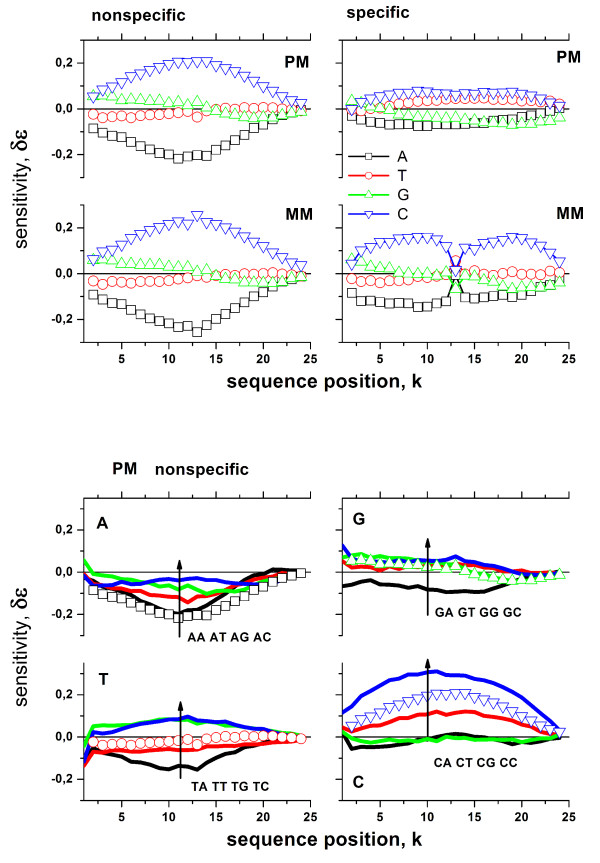
Positional dependent sensitivity profiles: The upper panel shows the single-nucleotide (SN) profiles for the PM and MM for non-specific and specific hybridization. The profiles are calculated using the intensity data shown in Figure 5 (Eq. **(23)**). The lower panel shows the NN-profiles of the PM for non-specific hybridization and the respective SN-profiles (lines) which are re-plotted from the upper panel (open symbols). Typically the values increase according to BA BT BG BC (with B = A, T, G, C; see arrows in the figure).

Typical SN- and NN-sensitivity profiles of the PM and MM referring to the N- and S-subsets are shown in Figure 6. Note their different shape, especially that of the S-profiles of the MM with the typical "dent" in the middle of the sequence. It is caused by the mismatched base pairing in the specific duplexes of the MM which give rise to changed interaction characteristics compared with WC-base pairings. We refer to our previous papers for the detailed discussion of the sensitivity profiles in terms of base-pair interactions in the respective probe-target duplexes [15,16,18]. In the present context it is important to discriminate between the four different sensitivity profiles to properly correct the intensity data for sequence specific effects.

Importantly, for perfect selection of the N-subset one expects the same sensitivity profiles for the PM and MM probes, since the nonspecific background bears no particular relationship to any of both kinds of probes. The profiles shown in Figure 6 confirm this prediction. The degree of similarity of the N-profiles of the PM and MM probes thus provides a criterion for the proper selection of the N-subsets.

### The corrected hook curve

In the next step, the corrected intensity values were transformed into (Δ_0,p_, Σ_0,p_)-coordinates using the corrected intensities in Eq. (9). Subsequently the (Δ_0,p_, Σ_0,p_)-data were set-averaged and smoothed in the same fashion as described above for the non-corrected intensities to get the sensitivity-corrected version of the hook-curve with the coordinates (Σ_0_^hook^, Δ_0_^hook^).

**Figure 7:**
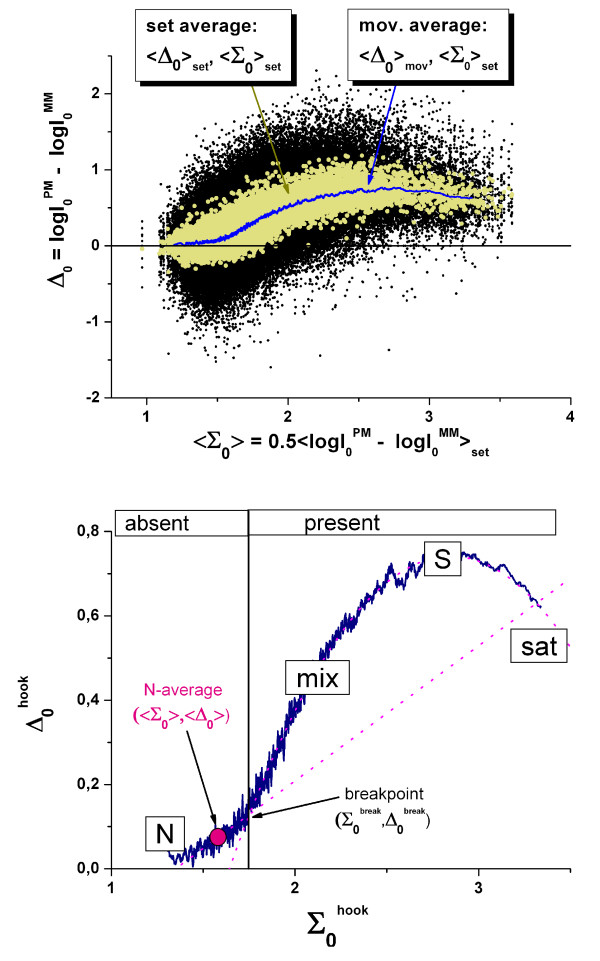
The same as Figure 5 but for sensitivity-corrected intensity data (Eq.(22)). The upper panel shows the Δ-vs-<Σ >_set _plot of the sensitivity-corrected intensity data of one chip taken from the HG-U133 spiked-in experiment (Affymetrix) The lower panel shows the (corrected) hook-curve.

The sensitivity-correction reduces the scattering of the probe- and probe-set-level intensity data about the smoothed hook-curve (see Figure 7). With the N-, mix-, S- and sat-hybridization regimes it essentially shows the same features as the uncorrected hook curve. The break between the N- and mix-regimes was again used to classify the probe sets into absent and present ones. The sensitivity correction affects the (Σ_0_^hook^, Δ_0_^hook^)-coordinates of each probe set with possible consequences for its classification into absent and present ones. In a second iteration we therefore re-calculated the four sensitivity profiles on the basis of corrected hook-curve by applying the same criteria for probe selection as above, i.e., Σ_0_^hook ^< Σ_0_^break ^and Σ_0_^hook ^> Σ_0_^80% ^for the N- and S-profiles, respectively. Typically the re-calculated sensitivity profiles only marginally change compared with the profiles which were obtained on the basis of the uncorrected hook curve indicating the relative robustness of the chosen classification criteria.

The detailed comparison between both versions of the hook-curve reveals subtle differences especially of the initial N-regime: After correction it changes slope and, more importantly, considerably narrows in horizontal direction by roughly 50% (compare Figure 7 and Figure 5). The steeper slope of the N-region after correction reflects the reduced cross correlation between the corrected PM- and MM-probe level data (see appendix B). Typically, the coefficient of correlation decreases from values *ρ *≈ 0.7–0.9 of the uncorrected data to *ρ *≈ 0.4–0.7 after correction which gives rise to the change of the slope by the factor of 1.5 – 3 (see Eq. (49)).

The narrowing of the N-range results from the partial removal of sequence effects which, to a large degree, cause the variability of probe signals [14]. Ideally, the correction of the intensities for sequence specific effects is expected to shrink the N-region into one point referring to one and the same corrected background intensity for all probes of the chip (see Eqs. (11) and (3)). The residual width of the N-region reflects the deficiency of the method due to at least three, essentially undistinguishable effects: Firstly, the "fit"-error of the position-dependent sensitivity model which only incompletely corrects the intensity data for sequence effects caused, e.g. by the specific folding of probe or target and/or bulk dimerization between different targets [1]; secondly, the "N-concentration" error due the simple assumption to consider all non-specific transcripts as one effective species (Eq. (3)) and thirdly, the "classification" error of the simple break-criterion which imperfectly distinguishes between "present" and "absent" probes due to its limited specificity.

### Fit of the hybridization model

**Figure 8:**
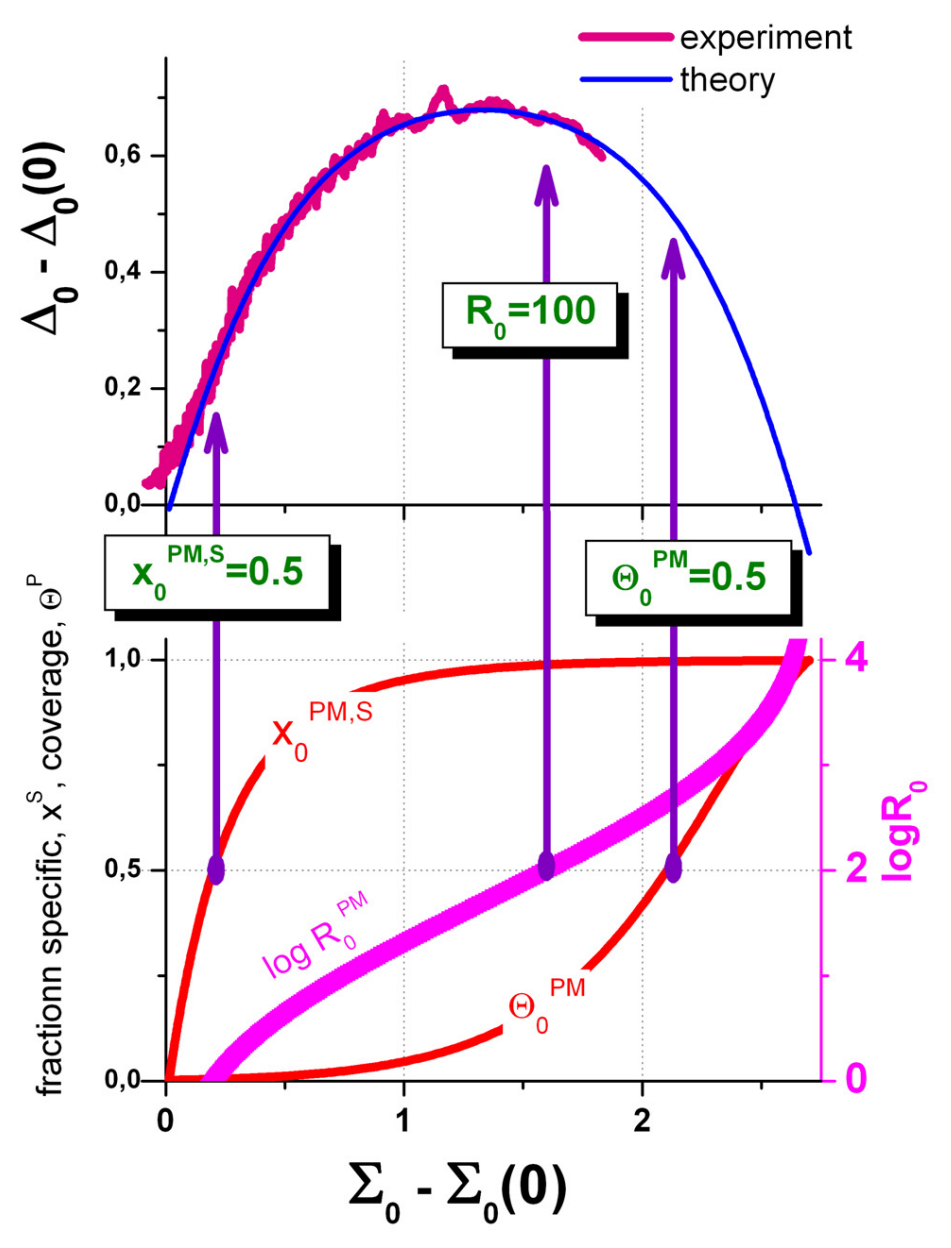
Fit of the Langmuir-model (Eq. **(10)**) to the mix-, S- and sat-ranges of the corrected hook curve (see Figure 7). The lower panel illustrates the relation between the coordinate axes and intrinsic probe characteristics: The respective fraction of specifically hybridized PM-probes (Eq. **(45)**), their S/N-ratio (Eq. **(46)**) and occupancy (Eq. **(43)**). The arrows indicate the 50%-conditions at which the PM-probes on the average become equally hybridized with specific and non-specific transcripts (x^PM, S ^= 0.5) and at which 50% of the oligomers of the PM-probe spots become occupied (Θ^PM ^= 0.5). The third arrow points to the probes with a 100fold excess of specific hybridization.

The corrected hook curve manifests the mean hybridization characteristics of the probes on the particular chip in sensitivity-corrected (Σ_0_^hook^, Δ_0_^hook^)-coordinates. It shows essentially the same properties as the Σ-vs-Δ trajectory of a single probe except the N-regime. For quantitative analysis we fit the hybridization model introduced above (Eq. (10)) to the corrected hook data in the mix-, S-, sat- and as-ranges (i.e. at Σ_0_^hook ^> Σ_0_^break^, see Figure 8). The least-squares gradient descent algorithm searches for optimum values of *α*_c_, *β*_c _and Σ_c_(0) ≈ Σ_0_^break ^and Δ_c_(0) ≈ <Σ_0_^hook^>_p∈N_. Note that the break position Σ_0_^break ^slightly deviates from the centre of the N-range <Σ_0_^hook^>_p∈N _because of the width of the N-regime (see Figure 7). We define the total width of the hook curve as $\beta_{c}=\Sigma_{c}\left(\infty \right)-{\langle \Sigma_{0}^{hook}\rangle }_{p\in N}$.

Note that the obtained model parameters are now chip-averaged mean values in contrast to the single probe properties used above. We therefore substitute the probe-index "p" by the chip-index "c". Particularly, one gets the mean PM/MM-ratios of the binding constants for specific and non-specific hybridization

(24) $$\begin{array}{ccc} {\text{s}}_{\text{c}}\equiv \frac{{\text{K}}_{\text{c}}^{\text{PM},\text{S}}}{{\text{K}}_{\text{c}}^{\text{MM},\text{S}}}\approx 10^{\alpha_{\text{c}}+\Delta_{\text{c}}(0)} & \text{and} & {\text{n}}_{\text{c}}\equiv \frac{{\text{K}}_{\text{c}}^{\text{PM},\text{N}}}{{\text{K}}_{\text{c}}^{\text{MM},\text{N}}}\approx 10^{\Delta_{\text{c}}(0)} \end{array},$$

respectively, and the mean binding strength for non-specific binding of the particular chip,

(25) $$\log{\text{X}}_{\text{c}}^{\text{PM},\text{N}}\approx -\beta_{\text{c}}+\frac{1}{2}\log{\text{n}}_{\text{c}},$$

in analogy with Eqs. (4) and (11) – (12). Here the "height" and "width" parameters, *α*_c _and *β*_c_, characterize the vertical and horizontal dimensions of the corrected hook curve of chip "c" (see Figure 7 and Figure 8) which in turn are related to the mean binding characteristics averaged over all probes of the chip, i.e., $\log{\text{K}}_{\text{c}}^{\text{P},\text{h}}\approx {\langle \log{\text{K}}_{\text{p}}^{\text{P},\text{h}}\rangle }_{\text{chip}}$ (P = PM, MM, h = N, S). The sensitivity-corrected S/N-ratio

(26) $$\text{R}\equiv {\text{R}}_{\text{set}}^{\text{PM}}=\frac{{\text{X}}_{\text{set}}^{\text{PM},\text{S}}}{{\text{X}}_{\text{c}}^{\text{PM},\text{N}}}=\frac{{\left[\text{S}\right]}_{\text{set}}}{{\left[\text{N}\right]}_{\text{c}}}\cdot \frac{{\text{K}}_{\text{c}}^{\text{PM},\text{S}}}{{\text{K}}_{\text{c}}^{\text{PM},\text{N}}},$$

scales the concentration ratio of specific and non-specific transcripts with the respective ratio of chip-averaged mean binding constants of the PM for specific and non-specific binding. Note that this scaling is common for all probes of the chip in contrast to the probe-specific scaling of R_p_^PM ^in Eq. (6).

In summary, the hook-curve analysis of the intensity data thus provides chip characteristics such as the mean contribution of the non-specific background, the asymptotic intensity maximum and the mean PM/MM-sensitivity gain, and, in addition, the expression degree on probe-set basis in terms of the S/N-ratio.

### Signal distributions

**Figure 9:**
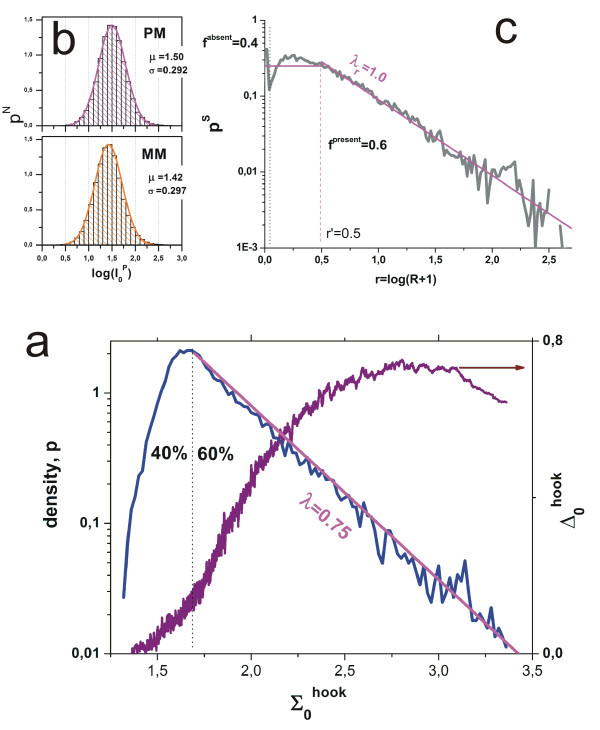
Probe-density distribution of the hook-curve (part a), the log-intensity distributions of the PM- and MM-probes taken from the N-range of the hook (part b) and the distribution of the S/N-ratio showing the specific signal distribution for log(R+1) > 0 (part c). About 40% of the probe pairs are absent according to the break-criterion. The distributions of their corrected intensities (bars) are well approximated by Gaussian functions (lines) with slightly shifted centres *μ *and nearly equal widths, *σ *(part b). Part c rescales the total distribution by using the argument r = log(R+1). The fraction of absent probes refers to intersection with the ordinate at r = 0 (40%). 60% of the probes are consequently called present with specific signals r > 0. Their distribution follows an exponential decay for values r ≥ r' = 0.5 with the decay constant *λ*_r_. In the intermediate range r' < r < 0 the signal is approximated by a constant (see Eq. **(28)**). The rescaling from Σ_0_^hook ^to log(R+1) slightly increases the decay constant (compare part b and a).

Part a of Figure 9 shows the probability-density distribution to find a probe pair a at a given position of the hook-abscissa, p(Σ_0_^hook^) = ΔN/(N_tot_·ΔΣ^hook^·) (here ΔN/N_tot _is the fraction of probe-pairs found per abscissa interval ΔΣ^hook^). About 40% of the probes fall in the N-range in this example and thus are classified "absent". We separately plot the density distribution of these absent probes as a function of their corrected log-intensities (part b of Figure 9). The obtained PM- and MM-probe-level data are well described by normal distributions (P_p_^N^(x, *σ*_c_) = N(*μ*_c_, *σ*_c_) with $x\equiv x_{p}={\left(\log I_{0,p}^{P}-\mu_{c}^{P}\right)}_{p\in N}$) of with virtually the same width (*σ*^PM ^≈ *σ*^MM^) and slightly shifted centres, $\mu_{\text{c}}^{\text{PM}}>\mu_{\text{c}}^{\text{MM}}(\mu_{c}^{P}={\langle \log I_{0,p}^{P}\rangle }_{p\in N})$.

One obtains the coordinates of the centre of the N-region of the hook-curve (see also Eq. (11)),

(27) $$\begin{array}{ccc} {\langle \Sigma_{0}\rangle }_{p\in N}=\mu_{p}^{PM}-\frac{1}{2}\log n_{c} & and & {\langle \Delta_{0}\rangle }_{p\in N}\equiv \log n_{c}=\mu_{c}^{PM}-\mu_{c}^{MM} \end{array}.$$

The total distribution decays exponentially to a good approximation at Σ_0_^hook ^> Σ_c_(0) in many cases (part a of Figure 9). Define the parameter $r\equiv \log X_{set}^{PM}-\mu^{PM}=\log(R+1)$, related to the S/N ratio R.

The exponential behaviour is approximately preserved with respect to this parameter in the mixed and S ranges since $r\approx \left(\Sigma^{hook}-\Sigma_{c}(0)\right)+\frac{1}{2}\left(\Delta^{hook}-\Delta_{c}(0)\right)$ from Eq. (18) (see part c of Figure 9). Note that r directly relates to the binding strength of specific hybridization in the relevant range of intermediate and large R-values, r ~ logR ~ log(X_set_^PM,S^). The initial value of the distribution at r = R = 0 provides the fraction of virtually absent probes, f^absent^, whereas the area under the normalized distribution for r > 0 consequently gives the fraction of present probes, f^present ^= (1 - f^absent^).

The distribution can be empirically approximated by an exponential decay function for r-values greater than a certain threshold r' (and R' > R)

(28) $$p_{c}^{S}(r,\lambda )\approx \{\begin{array}{ll} f_{1}^{present}\cdot \frac{\ln10}{\lambda_{r}}\cdot 10^{-(r-r^{\prime })/\lambda_{r}}; & r>r^{\prime }\\ f_{2}^{present}/r^{\prime }; & 0<r\leq r^{\prime }\\ f^{absent}\cdot \delta (r,0); & r\leq 0 \end{array}.$$

In the intermediate range the signal is approximated by a constant with f_2_^present ^= 1 - (f^absent ^+ f_1_^present^) (f^present ^= f_1_^present ^+ f_2_^present^) whereas for r = 0 one gets the fraction of absent probes. The decay constant *λ*_r _defines the r-range which decreases the probability of the specific signal by one order of magnitude. It thus defines a characteristic S/N-ratio (in logarithmic scale) of the chip which characterizes log-ratio of the mean specific and non-specific signals, or in other words, to which extend the specific signal exceeds the non-specific one. The S(pecific)/N(onspecific)-ratio thus can be also interpreted as a sort of S(ignal)/N(oise)-ratio of a given probe set. The value of the r-related decay constant *λ*_r _slightly exceeds that of the hook-related distribution owing to the larger range covered by the PMonly S/N-ratio (*λ*_r _= 1.0 versus *λ *= *λ*_Σ _= 0.75; compare part c and a of Figure 9).

An estimate of the mean decay constant can be obtained by simple averaging over the respective R-range

(29) ⟨*λ*⟩ ≈ (⟨log(*R *+ 1)⟩_*R *> *R*'_·In 10)

Note that the exponential decay in Eq. (28) is equivalent with the power law, 10^-r/*λ*r ^= (R+1)^-1/*λ*r^, which has been shown to describe the probability distribution of expression values in a series of microarray experiments [28-31]. This power-law function is known as the Zipf's law, observed in many natural and social phenomena. The presence of such power-law function in principle prevents an intrinsic cut off point between "on" genes and "off" genes. The analysis of expression data in terms of their probability distribution therefore is of basic importance for judging the expression level in terms of global characteristics. The hook-transformation provides such characteristics in terms of the signal distribution along the Σ^hook ^and/or r coordinates for the mix-, S- and sat-hybridization ranges.

Let us now consider the S/N-ratio of a particular probe at two conditions: (i) with x = 0, referring to the non-specific binding strength in the centre of the normal background distribution, X_p_^PM,N ^≈ 10^*μ*^/M_c_:$R_{p}=M_{c}\cdot X_{p}^{PM,S}/10^{\mu }$; and (ii) with x ≠ 0, i.e. X_p_^PM,N^(x) ≈ 10^*μ*+x^/M_c _and $R(x)=M_{c}\cdot X_{p}^{PM,S}/10^{x+\mu }$.

After transformation into logarithmic scale and combination of both situations we get log *R*(*x*) = log *R*_*p *_- *x *and for R > 1 to a good approximation *r*(*x*) ≈ *r *- *x*.

Now we combine the virtually independent background and signal distributions into the joint probability density function

(30) $$p_{p}^{P}(x,r-x)=p_{p}^{N}(x,\sigma^{P})\cdot p_{c}^{S}(r-x,\lambda ).$$

It refers to the logarithmic signal value r with the particular non-specific background contribution *μ *+ x. Integration over the whole possible backgound range provides the probability for detecting the signal r for probe p,

(31) $$P_{p}^{P}(r)=\int_{-\infty }^{\infty }p_{p}^{P}(x,r-x)\cdot dx.$$

Note that the upper integration range is effectively restricted to x ≤ r because of p^S^(r-x,*λ*) = 0 for x > r (see Eq. (28)). Eqs. (30)–(31) define the convolution product of the background and specific-signal distributions. A similar approach was used in ref. [30] and partly also in the RMA-algorithm [29].

### De-saturation of the signal and convolution based expression estimates

The hyperbolic intensity-response (Eq. (1)) can be linearized and transformed into the total binding strength using the asymptotic value M_c_:

(32) $$\begin{array}{ccc} L_{0,P}^{P}=\frac{I_{0,p}^{P}}{1-M_{c}^{-1}\cdot I_{0,p}^{P}} & and & X_{p}^{P}=\frac{L_{0,p}^{P}}{M_{c}} \end{array}.$$

**Figure 10:**
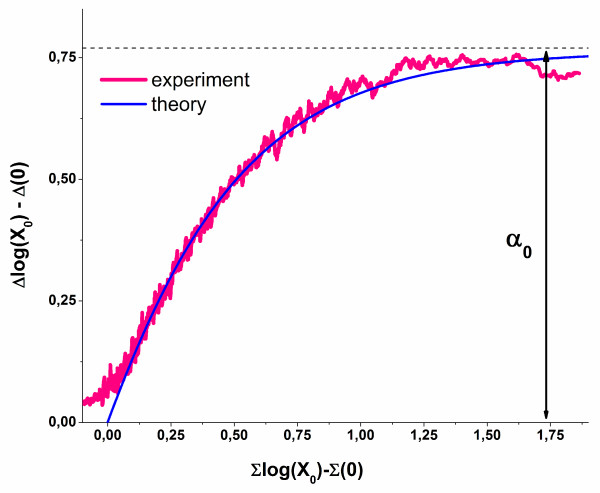
„Desaturated” hook-curve: The corrected intensities (see **Figure 7**) are linearized using Eq. **(32)**, transformed into Δ and Σ coordinates (Eq. **(9)**) and smoothed. After "linearization" the sat-range of the hook levels off into the asymptote of height *α *(Eq. **(24)**) which defines the PM/MM-gain upon duplexing (compare with **Figure 8**). The fit neglects the saturation terms in Eq. **(10)**.

Figure 10 re-plots the hook curve shown in Figure 8 after linearization of the corrected intensities using Eq. (32). The sat-range now levels off into the asymptotic value *α*_c _(compare with Figure 8). This "de-saturated" signal additively decomposes into contributions due to non-specific and specific hybridization (Eq. (2)). We now calculate the expression value as the weighted "glog"-mean of the specific signal,

(33) $$Z_{p}^{P}=\int_{-\infty }^{\infty }p_{p}^{P}(x,r-x)\cdot g\log\left(L_{0,p}^{P}-L_{p}^{P,N}(x,\mu_{c}^{P})\right)\cdot dx.$$

Particularly, Eq. (33) corrects the linearized signal (Eq. (32)) for the probe-specific background contribution using the mean background of the chip and the probe-specific sensitivity for non-specific hybridization, $L_{p}^{P,N}(x,\mu_{c}^{P})=10^{\delta A_{p}^{P,N}+\mu_{c}^{P}+x}$ (Eq. (23)). The use of the generalized logarithm, $\text{g}\log(\text{x})\equiv \log\left(\frac{1}{2}(\text{x}+\sqrt{{\text{x}}^{2}+\text{c}})\right)$, for data transformation is advantageous in two respects: Firstly, it enables processing negative arguments which may appear due to data scattering and, secondly, it "stabilizes" the variance at small values of x provided that the transformation parameter c > 0 is properly chosen [32,33].

In Eq. (33) the glog-transformed data are averaged over the probability distribution $p_{p}^{P}(x,r-x)$ representing the convolution product of the mutually independent probabilities of the non-specific Gaussian background and the specific expression signal (see previous section and [30]). This approach assumes that the probability distribution of each individual transcript is the same as the mean distribution averaged over all transcripts. Alternatively one can use the un-weighted kernel with p^S ^= const. In this case Eq. (33) restricts the averaging to the background distribution.

In the next step, the obtained values are corrected for the sequence effects according to $Z_{0,p}^{P}=Z_{p}^{P}-\delta A_{p}^{P,S}$ using the positional-dependent sensitivity model and the Z_p_^P ^values instead of the log-intensities for parameterization (Eq. (50)). Then, the probe-level data are summarized for each probe set by calculating the Tukey-biweight median, $Z_{0,set}^{P}=TB{\left(Z_{0,p}^{P}\right)}_{p\in set}$[25], and finally by transforming them into linear scale to get the probe set-level expression degree, $L_{set}^{P,S}=10^{Z_{set}^{P}}$. Eq. (33) thus provides "PMonly" and "MMonly" estimates of the expression degree with P = PM and MM, respectively.

Considering the fact that the MM intensity comprises information about the non-specific background one can correct the linearized intensity and background of the PM by subtracting that of the respective MM:

(34) $$Z_{p}^{PM-MM}=\int_{-\infty }^{\infty }p_{p}^{PM}(x,r-x)\cdot g\log\left(\Delta L_{0,p}-\Delta L_{p}^{N}(x,\mu_{c}^{P})\right)\cdot dx,$$

with $\Delta L_{0,p}=\left(L_{0,p}^{PM}-L_{0,p}^{MM}\right)$ and $\Delta L_{p}^{N}(x,\mu_{c}^{P})=\left(L_{p}^{PM,N}(x,\mu_{c}^{PM})-L_{p}^{MM,N}(x,\mu_{c}^{MM})\cdot 10^{-x\cdot (1-\rho )}\right)$

Eq. (34) uses the background distribution of the PM and the conditional expectation of the bivariate normal distribution for the argument of the MM-background which gives rise to an effectively shrunken integration variable, $\mu_{c}^{MM}+x\to \mu_{c}^{MM}+\rho_{c}x$ (here *ρ*_c _is the coefficient of correlation, see appendix B). Note that this approach is to some degree similar to the GC-RMA algorithm [34] which however uses pseudo-MM representing subsets of PM-probes of the same GC-content as the considered PM. A second difference to Eq. (34) is that GC-RMA directly subtracts the (non-linear) MM-intensity without explicit consideration of the non-specific background.

In this context we stress the fact that the non-specific background intensity is rather a variable contribution which progressively decreases with increasing amount of specific binding than a constant (see appendix A and the figure shown there). The background correction of the intensity (as realized, e.g. by GC-RMA) neglects this trend and therefore it is expected to over-correct the signal in the S- and as-hybridization ranges. As a consequence, this approach underestimates the expression degree due to two reasons: Firstly, this over-correction of the background and, secondly, the neglect of saturation. On the other hand, the linearized signal used here (Eq. (32)) corrects the intensity for saturation and, in addition, it contains a constant background level corrected by simple subtraction in Eqs. (33) and (34).

The joint PM-MM- and especially the MMonly-expression measures are smaller than the PMonly estimates because of the smaller binding constants of the MM for specific binding. The former two measures can be scaled to values equivalent to the expression level of the PM according to (see also Eq. (24)),

(35) $${\text{L}}_{\text{set}}={\text{L}}_{\text{set}}^{\text{PM},\text{S}}\approx {\text{s}}_{\text{c}}\cdot {\text{L}}_{\text{set}}^{\text{MM},\text{S}}\approx {\left(1-{\text{s}}_{\text{c}}^{-1}\right)}^{-1}\cdot {\text{L}}_{\text{set}}^{\text{PM}-\text{MM},\text{S}},$$

and finally transformed into the "set-averaged" binding strength in analogy with Eq. (32)

(36) $${\text{S}}_{\text{set}}={\text{X}}_{\text{set}}^{\text{PM},\text{S}}=\frac{{\text{L}}_{\text{set}}}{{\text{M}}_{\text{c}}}.$$

Note that the PM-estimate exceeds the MM signal roughly by the factor s_c _≈ 5 – 10 at comparable variance of the residual background distribution (see Figure 9, part b). For the coefficient of variation, CV ≈ *σ*/S, one gets roughly CV(PM) ≈ CV(PM-MM) << CV(MM). The MM consequently are expected to provide considerably less accurate expression estimates compared with the respective PMonly and PM-MM values.

Note that also the S/N-ratio (Eq. (26)) transforms into an alternative estimate of the specific binding strength of the PM (see Eq. (25) and (26)) with

(37) $${\text{S}}_{\text{set}}=\text{R}\cdot {\text{X}}_{\text{c}}^{\text{PM},\text{N}}=\text{R}\cdot 10^{-\beta_{\text{c}}+\frac{1}{2}\log{\text{n}}_{\text{c}}}.$$

Finally, the decay constant of the distribution of the specific signal relates to the logarithm of the S/N-ratio (see Eqs. (28) and (29)). One obtains the mean specific signal measured by the given chip as

(38) $$\begin{array}{ccc} {\langle S\rangle }_{c}=10^{-\varphi_{c}} & with & \varphi_{c}\equiv \left(\beta_{c}-\frac{1}{2}\log n_{c}\right)-\lambda_{r} \end{array}.$$

The characteristic expression index (or exponent of specific binding) *φ*_c _complements the respective exponents for non-specific hybridization *β*_c _and the characteristic S/N-index *λ*_r _as the basic chip summary-characteristics of specific and non-specific binding.

### Chip characteristics and expression estimates in natural units

Eqs. (36) and (37) provide probe set-estimates of the specific binding strength as a measure of the expression degree,

(39) $${\text{S}}_{\text{set}}\equiv {\text{X}}_{\text{set}}^{\text{PM},\text{S}}={\left[\text{S}\right]}_{\text{set}}\cdot {\text{K}}_{\text{c}}^{\text{PM},\text{S}},$$

which represents a dimensionless concentration measure in units of the mean specific binding constant of the chip. A value of S_set _= 1 consequently defines the condition of half-coverage to a good approximation (see Eq. (7)). Analogously, the horizontal dimensions of the hook-curve provide the non-specific binding strength as a measure of the non-specific background in units of the respective binding constant (see Eq. (25)),

(40) $${\text{N}}_{\text{c}}\equiv {\text{X}}_{\text{c}}^{\text{PM},\text{N}}={\left[\text{N}\right]}_{\text{c}}\cdot {\text{K}}_{\text{c}}^{\text{PM},\text{N}}.$$

It specifies the mean occupancy of the probes in the absence of specific transcripts, Θ^PM^(0) ≈ X_c_^PM,N^.

Hence, the hook method measures both, the abundance of specific and non-specific transcripts in the hybridization solution in chip-related units such as the relative occupancy of the probe spots and the respective binding strengths.

The specific and non-specific signals are combined into the S/N-ratio, R (Eq. (26)), which provides the expression degree in units of the non-specific background contribution in the particular chip experiment. The S/N-ratio is directly related to the hook-coordinates of a selected probe set and thus it can be roughly deduced by visual inspection of the particular hook curve (Eqs. (46) and (18)). Figure 8 illustrates the relation between the intrinsic expression measures and the hook coordinates for a typical microarray hybridization. The point of half coverage (Θ^PM ^= 0.5, X^PM, S ^≈ 1) is found beyond the maximum in the sat-regime. Virtually no probe of the chosen chip meets this condition. Note that Θ^PM ^scales with the distance relative to the end point referring to R → ∞ (see above). Vice versa, the mean fraction of specifically hybridized oligomers, x^PM, S^, scales with the distance relative to the N-point. It steeply increases in the mix-regime and reaches the conditions at which 50% of the bound transcripts belong to the specific ones, x^PM, S ^= 0.5, at relatively small abscissa-values. Hence, specific hybridization starts to dominate over non-specific one always at the beginning of the mix-range. The S-range near the maximum of the hook-curve refers to probes with a 50 – 100 fold excess of specific hybridization, R^PM ^≈ 50 – 100. The width of the hook of about *β *= 2.7 is equivalent with the background strength of N_c _≈ 10^-2.7 ^= 0.002 which in turn rescales the S/N-ratio into binding strengths, for example S_c _= 0.1–0.2 for R = 50–100. These rough estimation shows that the maximum of the hook is equivalent with the occupancy of the PM-spots in the order of 10 – 25%.

The mean binding constants, K_c_^PM,S ^and K_c_^PM,N ^and thus also the used measures of specific and non-specific hybridization depend on the particular probe and chip design (e.g., the length of the probe-oligonucleotides, their density and the type of the mismatches used for the MM probes). Consequently they are specific for the used chip type, on one hand. On the other hand, also the conditions of a particular hybridization affect the K_c_^PM,h ^because their values depend on all processes affecting the binding reaction between the probe-oligonucleotides and the targets. For example, the composition of a particular sample will affect K_c_^PM,N ^and K_c_^PM,S ^as well, because both constants depend on the extent of target-dimerization which is a function of the concentrations of the reacting species in the hybridization solution [1].

## 4. Summary and Conclusion

The improvement of microarray calibration methods in combination with the development of meaningful quality standards is an essential prerequisite for obtaining absolute expression estimates which in turn are required for the quantitative analysis of transcriptional regulation. In this publication we present a new method of microarray data analysis based on a physical model. This so-called hook method pre-processes the raw intensity data for further downstream analyses on one hand, and, on the other hand, provides chip characteristics with potential applications in hybridization quality control and array normalization.

**Figure 11:**
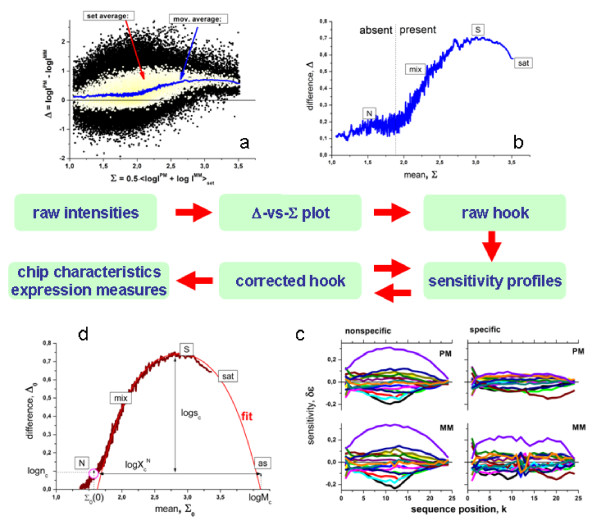
Schematic summary of the hook-method: The raw intensity data of one GeneChip microarray are plotted into the Δ = log(PM/MM)-vs-Σ = 1/2·log(PM·MM) coordinate system and smoothed to get the raw hook-curve. Then, probes from the N- and S-hybridization regimes are used to calculate four sets of position-dependent sensitivity profiles of the affinity model (non-specific and specific for the PM and MM each) which in turn are used to correct the intensities for sequence effects. The corrected intensities provide the corrected hook-curve. The mix-, S- and sat-ranges are fitted using the two-species Langmuir hybridization model. The dimensions of the hook, its width and height, provide hybridization characteristics of the chip which in turn are used to calculate probe-level expression measures.

The method is based on the Langmuir-hybridization model which provides a physically adequate and computationally feasible approach for microarray intensity calibration with the potency to improve existing methods. Our hook-calibration method uses this model together with the positional-dependent nearest-neighbour affinity correction. It is based on the linear transformation of the intensities of PM and MM probes from one chip into Δ-vs-Σ coordinates, probe-set averaging and smoothing. Here, the MM probes serve as an internal reference subjected essentially to the same hybridization law as the PM, however with modified characteristics. Figure 11 and Table 2 summarize the essential steps of the algorithm together with the output characteristics provided by the method.

**Table 2:** Hook method: algorithm and output characteristics

**Step**	**Output (chip and probe-set characteristics)**	**Eq.**
1) optical background correction using the Affymetrix zone-algorithm (see Ref. [26]).	Optical background (O, mean over all zones); the algorithm uses a scaled value with a scaling factor chosen between 0 and 1	(1)
2) Raw hook: Plot of the PM and MM probe intensity data into Δ-vs-Σ coordinates and smoothing over a sliding-window of ~100 probe sets. Classification into N- and S-probes using the breakpoint of the hook.	Raw hook curve	(9)
3) Parameterization of the positional dependent sensitivity-model separately for the PM and MM in the N and S-ranges and correction of the intensities for probe-specific sensitivities.	Sensitivity profiles (optional SN, NN or NNN models)	(22), (23); App. C
4) Corrected hook: re-iterate steps (2)–(3) with the corrected intensities to improve the sensitivity correction and the classification of the probes into absent and present ones	Corrected hook-curve Fraction of absent probes (%N), mean N-background level and width of the N-range	(27)
5) Fit of the hook-equation to the mix-, S-and sat-ranges of the corrected hook curve and analysis of the probe-level hook coordinates	Maximum intensity (M_c_), mean non-specific background level (N_c_^PM^), dimensions of the hook (*α*_c_, *β*_c_), PM/MM-affinity gain (s_c _and n_c_), parameters of the normal background distribution (*μ*_c_, *σ*_c_, *ρ*_c_) and of the signal distribution (*λ*), S/N-ratio (R), and occupancy (Θ) and fraction of specific binding (x^S^)	(10), (12), (24) – (26), (29); App. D, App. A
6) Calculation of probe-set related expression estimates (alternatively PMonly or PM-MM) by the joint processing of the intensity data and selected chip characteristics which corrects for the non-specific background, sequence-specific sensitivity and saturation	Expression measures (L_set_, S_set_)	(33) – (36)

The obtained hook-curve can be interpreted as a special representation of the binding isotherm where the explicit dependence of the probe intensities on the (usually unknown) transcript concentrations is replaced by the (experimentally available) relation between the PM- and the MM-probe intensities. It enables clear differentiation between different, well-defined regimes and it provides a set of chip summary characteristics which evaluate the performance of a given hybridization in terms simple parameters such as the mean non-specific background intensity, its saturation value, the mean PM/MM-sensitivity gain and the fraction of absent probes. The hook curve spans a natural metrics system for the expression estimates which reflects essential hybridization characteristics in terms of its geometric dimensions, width, height and "start"-coordinates.

The obtained single chip characteristics in combination with the sensitivity corrected probe-intensity values provide expression estimates scaled in natural units given by the binding constants of the particular hybridization. This way the method corrects the raw intensities for the non-specific background hybridization in a sequence-specific manner, for the potential saturation of the probe-spots with bound transcripts and for the sequence-specific binding of specific transcripts.

In the accompanying publication we illustrate the performance and potential applications in terms of quality control and expression analysis using a series of selected chip-types, hybridization conditions and benchmark experiments [19].

The beta-version of the hook-program can be downloaded from . The stand-alone JAVA program processes single-chips and chip-series in a batch-mode according to the scheme given in Figure 11 and Table 2. Chip and probe-set related characteristics such as expression degrees, hook-curves and sensitivity profiles are exported in html- as well as in tabular form and jpg-graphics.

## 5. Appendix

### A. Concentration scales of the Δ-vs-Σ-trajectory: Derivation of Eqs. (16) – (18)

The relative Σ- and Δ-coordinates of the log-transformed probe intensities with respect to its asymptotic value at R = ∞ directly rescale into the respective log-sum and -difference of the surface coverage of the PM and MM probes (see Eqs. (1) and (9))

(41) $$\begin{array}{c} \Sigma \log\Theta \equiv \frac{1}{2}\left(\log\Theta^{\text{PM}}+\log\Theta^{\text{MM}}\right)=\left(\Sigma -\Sigma \left(\infty \right)\right)\\ \Delta \log\Theta \equiv \log\Theta^{\text{PM}}-\log\Theta^{\text{MM}}=\left(\Delta -\Delta \left(\infty \right)\right) \end{array},$$

One obtains Eq. (16) for the mean coverage of the PM and MM with Eq. (41) and the definition, ⟨Θ⟩ ≡ 10^ΣlogΘ^. Rearrangement provides the surface coverage of the PM and MM probes as a function of the relative Σ- and Δ-coordinates,

(42) $$\begin{array}{ccc} \Theta^{\text{PM}}=10^{\left(\Sigma -\Sigma \left(\infty \right)\right)+\frac{1}{2}\left(\Delta -\Delta \left(\infty \right)\right)} & \text{and} & \Theta^{\text{MM}}=10^{\left(\Sigma -\Sigma \left(\infty \right)\right)-\frac{1}{2}\left(\Delta -\Delta \left(\infty \right)\right)} \end{array}.$$

The surface coverage splits into contributions due to non-specific and specific transcripts, Θ^*P *^= Θ^*P,N *^+ Θ^*P,S*^, with $\Theta^{P,h}=\frac{X^{P,h}}{1+X^{P}}$ (see Eq. (2)). Both contributions are functions of the composition of the hybridization solution expressed in terms of the S/N-ratio R (see Eq. (5)),

(43) $$\begin{array}{lll} \Theta^{PM,N}(R)=\frac{X^{PM,N}}{1+X^{PM,N}\cdot (1+R)} & and & \Theta^{PM,S}(R)=R\cdot \Theta^{PM,N}(R)\\ \Theta^{MM,N}(R)=\frac{X^{PM,N}/n}{1+X^{PM,N}\cdot (1/n+R/s)} & and & \Theta^{MM,S}(R)=R\cdot \frac{n}{s}\cdot \Theta^{MM,N}(R) \end{array}.$$

**Figure 12:**
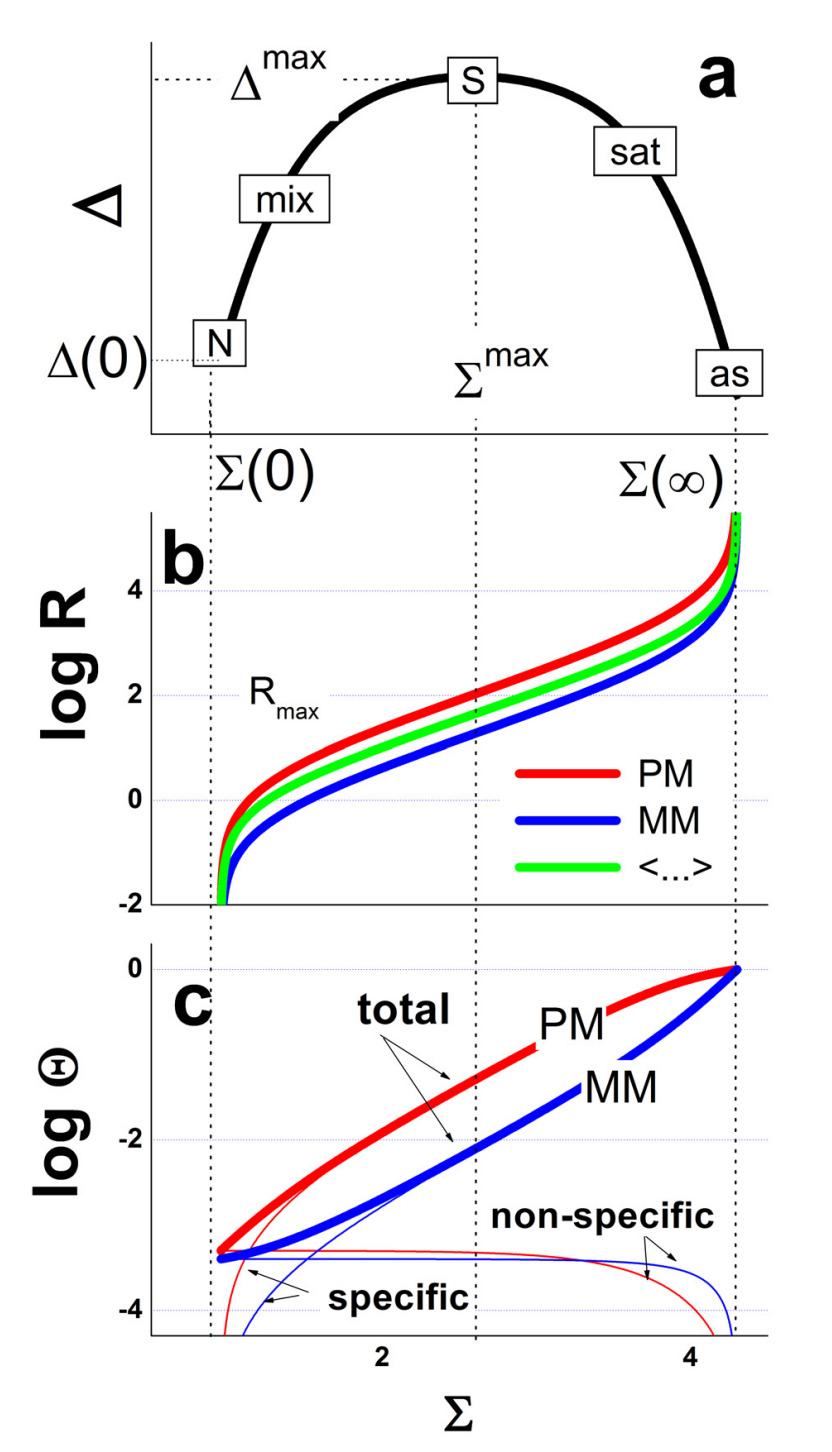
Δ-vs-Σ trajectory (part a), the S/N ratios of the PM, MM and of their mean (Eqs. **(46) **and **(18)**, part b) and the fraction of occupied PM and MM probes (occupancy, Eq. **(42)**, part c). The total occupancy additively decomposes into the occupancy due to specific and non-specific hybridization (thin lines, see Eq. **(43)**). Note that the latter occupancy is not a constant but it depletes upon increasing total occupancy because specific binding progressively replaces non-specific one.

Eq. (43) shows that the specific coverage monotonously increases with the S/N-ratio whereas the non-specific "background" coverage remains virtually constant at R < 1/X^PM, N ^for the PM (and at R < (n/(s·X^PM, N^) for the MM, see Figure 12). Specific transcripts of large binding strength progressively replace the bound non-specific fragments with further increasing values of R. As a consequence the non-specific coverage Θ^P, N ^drops and finally disappears for R → ∞.

With Eq. (43) one can express the specific and non-specific coverage as follows

(44) $$\begin{array}{ccc} \Theta^{\text{P},\text{S}}=\frac{\Theta^{\text{P}}-\Theta^{\text{P},\text{N}}(0)}{1-\Theta^{\text{P},\text{N}}(0)} & \text{and} & \Theta^{\text{P},\text{N}}=\Theta^{\text{P}}-\Theta^{\text{P},\text{S}} \end{array}.$$

The ratio of the "specific" and the total coverage defines the fraction of specifically hybridized probe-oligomers, ${\text{x}}^{\text{P},\text{S}}\equiv \frac{\Theta^{\text{P},\text{S}}}{\Theta^{\text{P}}}$ (see also Eqs. (43) and (44)). With Eqs. (44) and (42) one obtains,

(45) $$\begin{array}{ccc} {\text{x}}^{\text{PM},\text{S}}=\frac{1-10^{-\left\{\left(\Sigma -\Sigma (0)\right)+\frac{1}{2}\left(\Delta -\Delta (0)\right)\right\}}}{1-10^{-\beta -\frac{1}{2}\log\text{n}}} & \text{and} & {\text{x}}^{\text{MM},\text{S}}=\frac{1-10^{-\left\{\left(\Sigma -\Sigma (0)\right)-\frac{1}{2}\left(\Delta -\Delta (0)\right)\right\}}}{1-10^{-\beta +\frac{1}{2}\log\text{n}}} \end{array},$$

and Eq. (17) for the mean fraction, <x^S^>. Here we make use of $\Theta^{\text{PM},\text{N}}(0)=10^{-\beta -\frac{1}{2}\log\text{n}}$ and $\Theta^{\text{MM},\text{N}}(0)=10^{-\beta +\frac{1}{2}\log\text{n}}$ (see Eqs. (42) and (14)).

Finally, with ${\text{R}}^{\text{P}}\equiv \frac{\Theta^{\text{P},\text{S}}}{\Theta^{\text{P},\text{N}}}=\frac{{\text{x}}^{\text{P},\text{S}}}{1-{\text{x}}^{\text{P},\text{S}}}$ and Eq. (45) one obtains for P = PM, MM

(46) $$\begin{array}{ccc} {\text{R}}_{\text{p}}^{\text{PM}}=\frac{10^{\left\{\left(\Sigma -\Sigma (0)\right)+\frac{1}{2}\left(\Delta -\Delta (0)\right)\right\}}-1}{1-10^{\left\{\left(\Sigma -\Sigma (\infty )\right)+\frac{1}{2}\left(\Delta -\Delta (\infty )\right)\right\}}} & \text{and} & {\text{R}}^{\text{MM}}=\frac{10^{\left\{\left(\Sigma -\Sigma (0)\right)-\frac{1}{2}\left(\Delta -\Delta (0)\right)\right\}}-1}{1-10^{\left\{\left(\Sigma -\Sigma (\infty )\right)-\frac{1}{2}\left(\Delta -\Delta (\infty )\right)\right\}}} \end{array},$$

and Eq. (18) for the respective average $\langle \text{R}\rangle \equiv 10^{0.5\left(\log{\text{R}}^{\text{PM}}+\log{\text{R}}^{\text{MM}}\right)}$.

### B. The slope of the hook-curve in the mix- and the N-ranges

The initial part of the hook curve roughly divides into two segments of different slope. We assume that the probes from probe-sets with abscissa positions to the left from the break, Σ^hook ^< Σ^break^, are predominantly hybridized with non-specific transcripts (N-range) whereas the probes from probe-sets with abscissa positions to the right from the break, Σ^break ^< Σ^hook^, in addition, bind a certain amount of specific transcripts (mix-range). The slope of the hook curve in this mix-range reflects the change of the hook coordinates with increasing concentration of specific transcripts. From Eq. (10) one gets after differentiation with respect to the S/N-ratio R in the limit of R → 0,

(47) $${\text{slope}(\text{mix})=\frac{\partial \Delta^{\text{hook}}}{\partial \text{R}}\cdot \frac{\partial \text{R}}{\partial \Sigma^{\text{hook}}}|}_{\text{R}\to 0}\approx 2\frac{\left(1-10^{-\alpha_{\text{c}}}\right)}{\left(1-10^{-\alpha_{\text{c}}}\right)}\approx \min(\ln10\cdot \alpha_{\text{c}},2).$$

The initial slope of the mix range essentially depends on the "height"-parameter *α*_c _and thus on the PM/MM-ratio of the specific binding constants (see Eqs. (13) and (4)).

The slope of the hook-curve in the N-range can be rationalized in terms of cross-correlations between the non-specific background signals of the PM and MM probes referring to "absent" specific transcripts, i.e. R = 0. The variances of the hook-coordinates are given by

(48) $$\begin{array}{l} \begin{array}{cc} \sigma_{\Delta }^{2}=\sigma_{\text{PM}}^{2}+\sigma_{\text{MM}}^{2}-2\cdot \sigma_{\text{PM},\text{MM}}\approx 2<\sigma^{2}>\cdot \left(1-\rho \right) & \text{and} \end{array}\\ \sigma_{\Sigma }^{1}=\frac{1}{4}\left(\sigma_{\text{PM}}^{2}+\sigma_{\text{MM}}^{2}+2\cdot \sigma_{\text{PM},\text{MM}}\right)\approx \frac{1}{2}<\sigma^{2}>\cdot \left(1+\rho \right)\\ \begin{array}{cccc} \text{with} & <\sigma^{2}>\equiv \frac{1}{2}\left(\sigma_{\text{PM}}^{2}+\sigma_{\text{MM}}^{2}\right) & \text{and} & \rho \equiv \frac{\sigma_{\text{PM},\text{MM}}}{\sigma_{\text{PM}}\cdot \sigma_{\text{MM}}}\approx \frac{\sigma_{\text{PM},\text{MM}}}{<\sigma^{2}>} \end{array} \end{array}.$$

Here *σ*_PM_^2^, *σ*_MM_^2 ^and *σ*_PM,MM _denote the variance and covariance of the set-averaged log-intensities of the PM and MM probes in the N-range. The formula for the correlation coefficient *ρ *assumes $<\sigma^{2}>\approx \sigma_{\text{PM}}^{2}\approx \sigma_{\text{MM}}^{2}$ to a good approximation (see Figure 9).

With Eq. (48) one obtains for the mean slope of the hook-curve in the N-range

(49) $$\text{slope}(\text{N})\propto \sqrt{\frac{\sigma_{\Delta }^{2}}{\sigma_{\Sigma }^{2}}}=2\sqrt{\frac{\left(1-\rho \right)}{\left(1+\rho \right)}}.$$

Hence, a tiny slope near zero indicates strongly correlated PM and MM signals (*ρ*~1) whereas the increase of slope(N) reflects "decoupling" of PM and MM intensities.

### C. Fit of the position-dependent sensitivity profiles

The sensitivity terms *δε*_k_^P,h^(b_m_) in Eq. (23) were estimated using the so-called probe sensitivity [14],

(50) $${\text{Y}}_{\text{p}}^{\text{P},\text{h}}(\exp)\equiv \log{\text{I}}_{\text{p}}^{\text{P}}-{\langle \log{\text{I}}_{\text{p}}^{\text{P}}\rangle }_{\text{set}\in \text{h}},$$

which normalizes the log-intensities with respect to their log-mean over the probe set. Here the probe set was selected from the specific or non-specific sub-ensembles (h = N or S). P = PM, MM specifies the probe type. Insertion of Eq. (22) into (50) provides

(51) $$Y_{\text{p}}^{\text{P},\text{h}}(\text{theory})\approx \delta {\text{A}}_{\text{p}}^{\text{P},\text{h}}-{\langle \delta {\text{A}}_{\text{p}}^{\text{P},\text{h}}\rangle }_{\text{set}\in \text{h}}=\sum_{\text{k}=1}^{25-\text{m}+1}\sum_{{\text{b}}_{\text{m}}}\left(\delta \varepsilon_{\text{k}}^{\text{P},\text{h}}\left({\text{b}}_{\text{m}}\right)\cdot \left(\delta_{\text{k}}\left({\text{b}}_{\text{m}},\xi_{\text{k},\text{m}}^{\text{P}}\right)-{\text{f}}_{\text{k}}\left({\text{b}}_{\text{m}}\right)\right)\right).$$

Here f_k_(b_m_) is the probability to find the subsequence b_m _at sequence position k within the probes of a probe set. The weighted least squares algorithm fits Y(theory) to Y(exp) by optimizing the coefficients *δε*_k_^P,h^(b_m_) using single value decomposition [35].

The NN model provides 16 profiles *δε*_k_^P,h^(BB') (k = 1...24) and the NNN model 64 profiles *δε*_k_^P,h^(BB'B") (k = 1...23) per sub-ensemble (N or S) and probe type (PM or MM). To extract the basic trends for a qualitative discussion we reduce the number of data simply by transforming the NN- and NNN-profiles into single-base (SN)-profiles by appropriate averaging

(52) $$\begin{array}{l} \varepsilon_{\text{k}}^{\text{P},\text{h}}(\text{B})=\frac{1}{2}\sum_{{\text{B}}^{\prime }=\text{A},\text{T},\text{G},\text{C}}\left(\varepsilon_{\text{k}-1}^{\text{P},\text{h}}({\text{B}}^{\prime }\text{B})+\varepsilon_{\text{k}}^{\text{P},\text{h}}(\text{B}{\text{B}}^{\prime })\right)\\ \varepsilon_{\text{k}}^{\text{P},\text{h}}(\text{B})=\frac{1}{3}\sum_{\left({\text{B}}^{\prime }=\text{A},\text{T},\text{G},\text{C})({\text{B}}^{\prime \prime }=\text{A},\text{T},\text{G},\text{C}\right)}\left(\varepsilon_{\text{k}}^{\text{P},\text{h}}(\text{B}{\text{B}}^{\prime }{\text{B}}^{\prime \prime })+\varepsilon_{\text{k}-1}^{\text{P},\text{h}}({\text{B}}^{\prime }\text{B}{\text{B}}^{\prime \prime })+\varepsilon_{\text{k}-2}^{\text{P},\text{h}}({\text{B}}^{\prime }{\text{B}}^{\prime \prime }\text{B})\right) \end{array}.$$

### D. Fit of the Langmuir model to the Δ-vs-Σ data

Rearrangement of the parametric equation for the abscissa of the hook-data (Eq. (10)) provides a quadratic equation for the S/N-ratio with the non-negative solution

(53) $$\begin{array}{l} \text{R}=-\frac{\text{p}}{2}+\sqrt{\frac{{\text{p}}^{2}}{4}+\text{q}}\geq 0\\ \text{p}=(1+10^{+\alpha_{\text{c}}})-\frac{10^{2\left(\Sigma_{0}^{\text{hook}}-\Sigma_{0}(0)\right)}}{\left(1-10^{-2\beta_{0}}\right)}10^{-\left(\beta_{\text{c}}+\frac{1}{2}\Delta_{\text{c}}(0)\right)}\left(10^{\Delta_{\text{c}}(0)}+10^{+\alpha_{\text{c}}}\right)\\ \text{q}=10^{+\alpha_{\text{c}}}\left\{\frac{10^{2\left(\Sigma_{0}^{\text{hook}}-\Sigma_{\text{c}}(0)\right)}}{\left(1-10^{-2\beta_{\text{c}}}\right)}\left(1+10^{-\left(\beta_{\text{c}}+\frac{1}{2}\Delta_{\text{c}}(0)\right)}\left(1+10^{\Delta_{\text{c}}(0)}\right)\right)-1\right\} \end{array}.$$

Equation (53) thus returns the S/N-ratio R for the Σ_0_^hook^-coordinate of a probe set and a given set of model-parameters {*α*_c_, *β*_c_, Σ_c_(0), Δ_c_(0)}. The parametric equation for the hook-ordinate (Eq. (10)) then provides an estimate of Δ_0_(R) referring to the respective probe set. The fit-algorithm minimizes the sum of least squares between the calculated and measured Δ-values, $\text{SSQ}=\sum_{\text{i}}{\text{w}}_{\text{i}}\cdot {\left(\Delta_{\text{i},0}({\text{R}}_{\text{i}})-\Delta_{\text{i},0}^{\text{hook}}\right)}^{2}$, by adjusting the parameters *α*_c_, *β*_c _and Σ_c_(0) using an iterative gradient method with linearly decreasing step size. The ordinate value of the start-point was set to the break between the N- and mix-range, $\Delta_{\text{c}}(0)=\Delta_{0}^{\text{break}}$ (see above).

We divide the Σ_0_^hook^-axis into i = 10 – 30 equally-spaced sampling points Σ_0_^hook ^= Σ_i _by averaging over the probe/probe set data within the respective sampling interval of width *δ*Σ, i.e., $\Delta_{\text{i},0}^{\text{hook}}=\langle \Delta_{\text{p},0}^{\text{hook}}\rangle$ for all probes with $\Sigma_{\text{i}}-\delta \Sigma \leq \Sigma_{\text{p},0}^{\text{hook}}<\Sigma_{\text{i}}+\delta \Sigma$ which were used in Eq. (53) to calculate the respective S/N-ratios R_i _and theoretical ordinate points Δ_i,0_(R_i_) using Eq. (10). The weighting factor, ${\text{w}}_{\text{i}}\approx \frac{\rho (\Sigma_{\text{i}})}{\sigma_{\Delta }{\left(\Sigma_{\text{i}}\right)}^{2}}$, considers the data-density, *ρ*(Σ_i_), and variance, *σ*_Δ_(Σ_i_)^2^, per interval. Both, *ρ*(Σ_i_) and *σ*_Δ_(Σ_i_)^2^, are given by decaying functions ([30,32]) which partly compensate each other to a good approximation. By default the weighting factor is therefore set to w_i _= 1.

## Competing interests

The authors declare that they have no competing interests.

## Authors' contributions

Both authors invented the method. HB leads the project and wrote the paper. SP wrote the computer program for data analysis and helped to draft the paper. Both authors read and approved the final manuscript.
